# Identification and Expression Patterns of *Anoplophora chinensis* (Forster) Chemosensory Receptor Genes from the Antennal Transcriptome

**DOI:** 10.3389/fphys.2018.00090

**Published:** 2018-02-13

**Authors:** Long Sun, Ya-Nan Zhang, Jia-Li Qian, Ke Kang, Xiao-Qing Zhang, Jun-Dan Deng, Yan-Ping Tang, Cheng Chen, Laura Hansen, Tian Xu, Qing-He Zhang, Long-Wa Zhang

**Affiliations:** ^1^Anhui Provincial Key Laboratory of Microbial Control, School of Forestry & Landscape Architecture, Anhui Agricultural University, Hefei, China; ^2^College of Life Sciences, Huaibei Normal University, Huaibei, China; ^3^Forest Diseases and Insect Pests Control and Quarantine Station of Chaohu City, Chaohu, China; ^4^College of Environmental Science and Forestry, State University of New York, Syracuse, NY, United States; ^5^Sterling International, Inc., Spokane, WA, United States

**Keywords:** antennal transcriptome, expression pattern, odorant receptor, gustatory receptor, ionotropic receptor, *Anoplophora chinensis*

## Abstract

The citrus long-horned beetle (CLB), *Anoplophora chinensis* (Forster) is a destructive native pest in China. Chemosensory receptors including odorant receptors (ORs), gustatory receptors (GRs), and ionotropic receptors (IRs) function to interface the insect with its chemical environment. In the current study, we assembled the antennal transcriptome of *A. chinensis* by next-generation sequencing. We assembled 44,938 unigenes from 64,787,784 clean reads and annotated their putative gene functions based on gene ontology (GO) and Clusters of Orthologous Groups of proteins (COG). Overall, 74 putative receptor genes from chemosensory receptor gene families, including 53 ORs, 17 GRs, and 4 IRs were identified. Expression patterns of these receptors on the antennae, maxillary and labial palps, and remaining body segments of both male and female *A. chinensis* were performed using quantitative real time-PCR (RT-qPCR). The results revealed that 23 ORs, 6 GRs, and 1 IR showed male-biased expression profiles, suggesting that they may play a significant role in sensing female-produced sex pheromones; whereas 8 ORs, 5 GRs, and 1 IR showed female-biased expression profiles, indicating that these receptors may be involved in some female-specific behaviors such as oviposition site seeking. These results lay a solid foundation for deeply understanding CLB olfactory processing mechanisms. Moreover, by comparing our results with those from chemosensory receptor studies in other cerambycid species, several highly probable pheromone receptor candidates were highlighted, which may facilitate the identification of additional pheromone and/or host attractants in CLB.

## Introduction

The citrus long-horned beetle (CLB), *Anoplophora chinensis* (Forster) (Coleoptera: Cerambycidae) is a polyphagous wood-boring beetle native to China, Japan, and the Korean peninsula (Haack et al., 2010). This beetle has spread to Europe through international shipments of wood-packing materials and live plants from Asia and is a quarantine pest species on the European Union (EU) and European and Mediterranean Plant Protection Organization (EPPO) A1 list (Rizzi et al., 2013; Ge et al., 2014). It has a very broad range of host plants (>100 species from 19 families), of which 48 species are distributed in China (Ge et al., 2014). Larval infestation damages the vascular system and woody tissues of host plants, ultimately causing severe damage to ornamental and forest trees that may lead to mortality (Haack et al., 2010). As in most insects, CLB utilizes olfaction to recognize volatile cues that regulate a series of behaviors including mating, foraging, oviposition, and host-seeking. Recently, Yasui and Fujiwara-Tsujii (2016) discovered the sesquiterpene β-elemene can function as a female-acquired repellant pheromone against males from a different host plant population in *Anoplophora malasiaca*, a synonym of *A. chinensis*, while Hansen et al. (2015) identified a male-produced pheromone component, 4-(*n*-heptyloxy)butan-1-ol for *A. chinensis*.

Peripheral olfactory proteins include odorant binding proteins (OBPs), chemosensory proteins (CSPs), odorant receptors (ORs), ionotropic receptors (IRs), gustatory receptors (GRs), and sensory neuron membrane proteins (SNMPs) (Leal, 2013). ORs, GRs, and IRs are membrane-bound chemosensory receptors localized to sensillum chemosensory dendrites, bridge the gap between the extracellular odorant signal and the intracellular neurological response, and are critical for the olfactory response (Xu et al., 2015). These receptors are particularly attractive molecular targets for the development of new pest control strategies. ORs are seven transmembrane domain proteins with an inverted membrane topology (Ha and Smith, 2009; Leal, 2013). A heterometeric ligand-gated ion channel between an olfactory receptor co-receptor (Orco) and a more specialized OR is required in order to transduce odor-evoked signals (Gu et al., 2015). Orco acts as an ion channel and is highly conserved across insect orders and widely expressed in the majority of ORNs (Leal, 2013; Cattaneo et al., 2017). More specialized ORs may be tuned to a pheromone, certain plant volatiles, or other compounds (Ha and Smith, 2009; Liu et al., 2013; Cattaneo et al., 2017). Insect GRs are mainly expressed in gustatory receptor neurons (GRNs) of the gustatory organs (Ebbs and Amrein, 2007; Crava et al., 2016), but are also found in ORNs (Scott et al., 2001). Most insect gustatory organs are distributed on body surfaces such as proboscises, legs, wings, female genitals, and labial palps (Scott et al., 2001; Vosshall and Stocker, 2007). These GRs generally detect soluble compounds acquired from contact with a substrate, including sugars, amino acids, salts, and bitter compounds, but can also respond to carbon dioxide or pheromone signals (Ebbs and Amrein, 2007; Kwon et al., 2007; Sánchez-Gracia et al., 2009; Zhang et al., 2013). Insect IRs are a novel family of chemosensory receptors that are related to ionotropic glutamate receptors (iGluRs) (Benton et al., 2009; Croset et al., 2010), and act as ligand-based ion channels (Croset et al., 2010; Abuin et al., 2011). IRs are a more ancestral and conserved group of receptors than ORs and have been identified throughout protostomes, including nematodes, arthropods, mollusks, and annelids (Croset et al., 2010; Gu et al., 2015; Wang et al., 2015). Insect IRs are generally divided into two subfamilies, “antennal IRs,” expressed in insect antennal ORNs, and species-specific “divergent IRs,” mainly expressed in the gustatory organs and involved in the detection of tastants (Croset et al., 2010). Two well-conserved antennal IRs, IR8a, and IR25a, have a similar function to Orco and are diffusely expressed in insect ORNs (Croset et al., 2010; Kaupp, 2010; Abuin et al., 2011). IRs are essential for odor-evoked neuronal responses and for detecting environmental volatile chemicals and tastes (Croset et al., 2010; Ai et al., 2013; Rytz et al., 2013).

The objectives of our study were to (1) identify the chemosensory receptors (ORs, GRs and IRs) of *A. chinensis* via the antennal transcriptome sequencing, (2) examine the expression profiles of these receptors in multiple tissues of both sexes using quantitative real time PCR (RT-qPCR), (3) conduct a thorough comparison to the ORs identified in other cerambycid species including *Megacyllene caryae* and *Anoplophora glabripennis*, which may contribute to the identification of additional pheromone and host attractants in CLB, and (4) compare and contrast *A. chinensis* ORs identified in our study to those recently identified by Wang et al. (2017). Although there is some overlap, the strong disparities in research priorities [olfactory binding-protein genes families (OBPs and CSPs) *vs*. chemosensory receptor superfamilies (ORs, GRs, and IRs)], insect samples (sample size, collection sites and host plants) and total number of receptor genes identified between these two studies (see discussion for a detailed comparison) make both works complementary and valuable, and cross-validate each other.

## Materials and methods

### Insects and tissue collections

Live adult CLBs were collected from *Acer rubrum* stands in Hefei, Anhui Province, China in June, 2017. Forest Pest Control Station of Anhui Province issued the permit for the field collection (by the director, Jun Fu). Beetles were sexed and reared separately on fresh shoots of *A. rubrum* in clean, well-ventilated plastic cages (17.0 × 12.0 × 6.8 cm) at 25°C and 75% RH. Excised female and male antennal tissues were immediately frozen in liquid nitrogen, and then stored at−80°C for subsequent RNA-seq sequencing.

### RNA extraction, cDNA library construction and illumina sequencing

The antennae of both sexes were blended for total RNA extraction using TRIzol reagent (Invitrogen, Carlsbad, CA, USA) according to the manufacturer's instructions. RNA degradation and contamination were monitored on 1% agarose gel, RNA concentration was measured using Qubit® RNA Assay Kit with a Qubit® 2.0 Fluorometer (Life Technologies, CA, USA), and RNA purity was evaluated with a NanoPhotometer® spectrophotometer (Implen, CA, USA). Illumina sequencing of the samples was performed at Novogene Co., Ltd., Beijing, China. Sequencing libraries were generated using NEBNext® Ultra™ RNA Library Prep Kit for Illumina® (NEB, Ipswich, MA, USA) according to manufacturer's recommendations, and index codes were added to attribute sequences to each sample. Briefly, mRNA was purified from total RNA using poly-T oligo-attached magnetic beads. Fragmentation was carried out using divalent cations under elevated temperature in 5X NEBNext First Strand Synthesis Reaction Buffer.

First strand cDNA was synthesized using random hexamer primer and M-MuLV Reverse Transcriptase (RNase H). Second strand cDNA synthesis was then performed using DNA Polymerase I and RNase H. Remaining overhangs were converted into blunt ends via exonuclease/polymerase activities. After adenylation of DNA fragment 3' ends, NEBNext Adaptor with hairpin loop structure was ligated to prepare for hybridization. The adaptor-ligated cDNA was incubated at 37°C for 15 min followed by 5 min at 95°C prior to PCR with Phusion High-Fidelity DNA polymerase, Universal PCR primers and Index (X) Primer. PCR products were purified (AMPure XP system) and library quality was assessed on the Agilent Bioanalyzer 2,100 system. Finally, library preparations were sequenced on an Illumina Hiseq™ 4,000 platform and paired-end reads were generated.

### Assembly and functional annotation

Clean reads were obtained from raw data by removing low quality reads and reads containing adapter or poly-N. A transcriptome was assembled based on clean reads using Trinity (Grabherr et al., 2011) to generate transcripts.

Unigenes were obtained from transcriptome assembly by choosing the longest transcript of each gene. BLASTx searches were used to align unigenes and compare them to the NCBI non-redundant (nr) protein database using an E-value threshold of 1 × 10^−5^. Unigenes were also annotated using other protein databases including Nt, Pfam, KOG/COG, Swiss-Prot, KO, and GO. ORFs of each unigenes were then predicted with ORF finder (http://www.ncbi.nlm.nih.gov/gorf/gorf.html) and the transmembrane domains of putative olfactory genes were determined using TMHMM Server v. 2.0 (http://www.cbs.dtu.dk/services/TMHMM/).

### Phylogenetic analysis

OR, GR, and IR amino acid sequences from *A. chinensis* and other insect species were aligned using ClustalX2.0. The OR data set contained identified sequences from *A. chinensis* (53), *Tribolium castaneum* (47), *A. glabripennis* (25), *M. caryae* (30), *Dendroctonus ponderosae* (12) (Andersson et al., 2013) and *Ips typographus* (19) (Andersson et al., 2013), along with 4 Orco genes from *Phyllotreta striolata* (Wu et al., 2016), *Anomala corpulenta* (Li et al., 2015), *Monochamus alternatus* (Wang et al., 2014) and *Tenebrio molitor* (Liu et al., 2015). The GR data set included 67 protein sequences reported from *Drosophila melanogaster* (7), and *Bombyx mori* (4), and the six coleopterans: *A. chinensis* (17), *P. striolata* (16), *A. glabripennis* (7), *T. castaneum* (11), *D. ponderosae* (2) and *I. typographus* (3) (Scott et al., 2001; Robertson et al., 2003; Wanner and Robertson, 2008; Guo et al., 2017). The IR data set contained sequences from *A. chinensis* (4), *A. glabripennis* (1), *P. striolata* (15), *M. alternatus* (6), *A. corpulenta* (5), *D. ponderosae* (10), *I. typographus* (4), *T. molitor* (6), and *D. melanogaster* (10). OR, GR, and IR unrooted phylogenetic trees were constructed using the MEGA6 neighbor-joining method (Tamura et al., 2013). Node support was assessed by bootstrap method with 1,000 bootstrap replicates.

### RT-qPCR validation of ORs, GRs, and IRs

Expression profiles of putative chemosensory receptor unigenes in different body sections of both sexes were analyzed with RT-qPCR using an ABI 7300 Real-Time PCR System (Applied Biosystems, Foster City, CA, USA). Total RNA was isolated from 20 antennae, 100 maxillary palps, 100 labial palps, and 20 bodies without antennae, maxillary palps, and labial palps from each sex, using the methods described above. Isolated RNA was reverse transcribed into cDNA using PrimeScript1 RT reagent Kit with gDNA Eraser (Perfect Real Time, Takara, Beijing, China). 2.5 ng cDNA was used as the RT-qPCR template. RT-qPCR target and reference gene primers were designed using Beacon Designer 7.9 software (PREMIER Biosoft International, Palo Alto, CA, USA) with CLB GAPDH (glyceraldehyde-3-phosphate dehydrogenase) and actin reference genes (Table S1). The RT-qPCR reaction mixtures were composed of 20 μL 2×SYBR Green qPCR Master Mix-R (YIFEIXUE BIO TECH, Nanjing, China), 0.4 μL of both forward and reverse primer (10 μM), 1 μL sample cDNA, and 8.2 μL sterilized H_2_O. RT-qPCR cycling parameters were set at 95°C for 10 min, followed by 40 cycles of 95°C for 15 s and 60°C for 1 min. The Q-gene method (Simon, 2003) was used to calculate the expression levels of these genes in the four tissues from each sex. RT-qPCR data were analyzed and plotted using Graphpad Prism 5.0 (GraphPad Software, CA, U.S.A).The statistical classification of each target gene was calculated in each tissue with SPSS 22.0 (SPSS Inc., Chicago, IL, USA) using a one-way nested analysis of variance (ANOVA) followed by Duncan's new multiple range test (α = 0.05).

## Results

### Transcriptome sequencing and unigenes assembly

In total, 66,908,284 raw reads and 64,787,784 clean reads with a Q20 percentage of 97.02% were obtained from the CLB antennal transcriptome. From these, 44,938 unigenes were screened from 89,311 transcripts. Unigene and transcript mean lengths were 1392 and 842 bp, respectively, while N50 lengths were 2143 and 1718 bp, respectively. Length distribution analysis indicated that 33,989 unigenes, or 75.63% of all unigenes, were longer than 500 bp and the longest unigene was 26,202 bp (Figure S1). 26,701 unigenes (59.41%) were compared to proteins in the NCBI non-redundant (nr) protein database using the BLASTX algorithm (cut-off E-value of 10^−5^). Homology analysis with other insect species revealed that *T. castaneum* was the best match (55.6%), followed by *D. ponderosae* (14.4%) and *Lasius niger* (1.8%) (Figure S2).

### Gene ontology (GO) annotation and KEGG analysis

**Figure 1:**
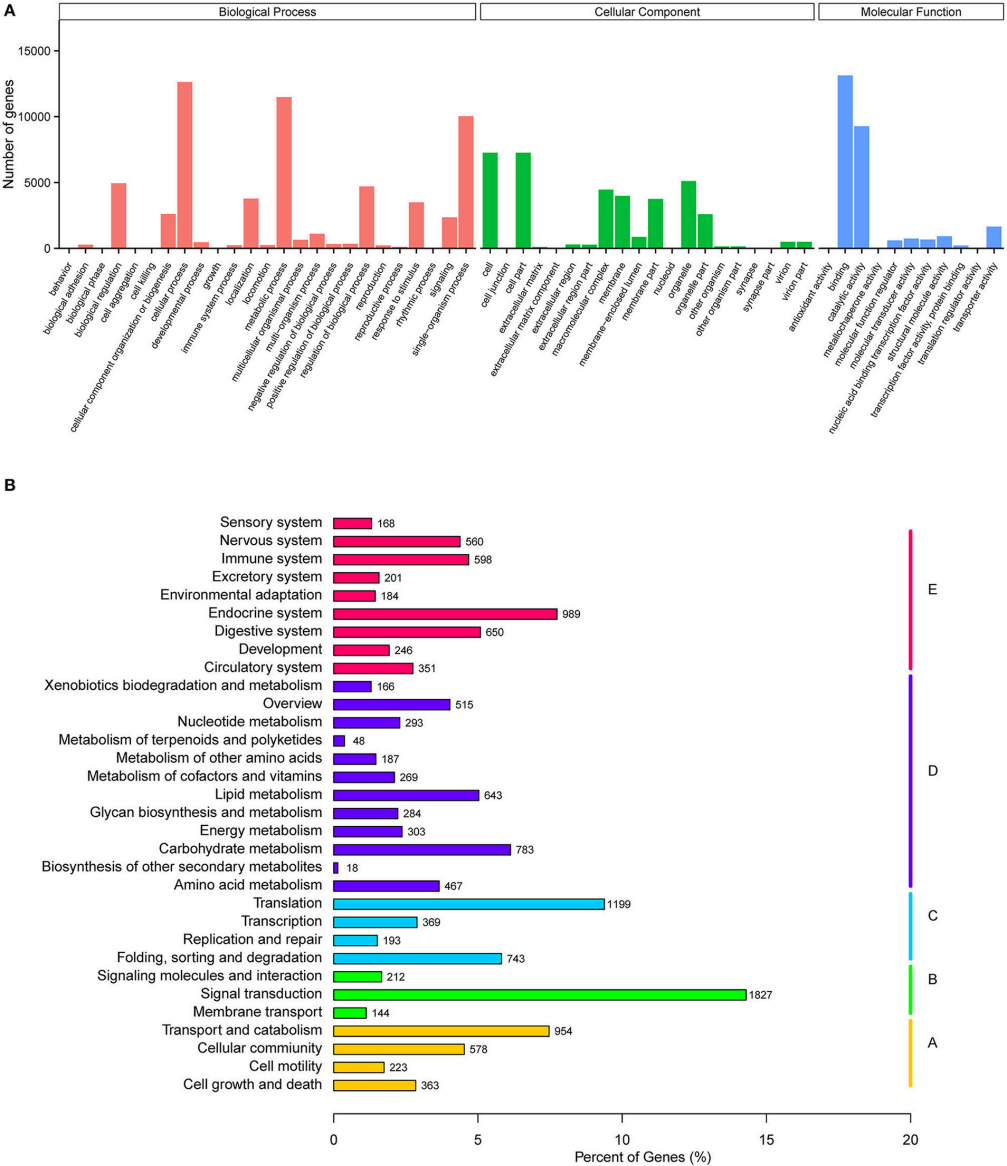
Functional annotation of *A. chinensis* unigenes. **(A)** Gene ontology (GO) classification of *A. chinensis* unigenes. **(B)** Kyoto Encyclopedia of Genes and Genomes (KEGG) classification of *A. chinensis* unigenes.

GO annotation was used to classify unigenes into different functional categories. Overall, Blast2GO (Götz et al., 2008) assigned 48.52% (21,808) of unigenes to three functional categories: cellular components (37,292), biological processes (60,089), and molecular function (27,255) (Figure 1A). In cellular components, cell part (7,260), cell (7,260), and organelle cellular component (5,113) were the most represented subcategories, in biological processes, cellular process (12,624), metabolic process (11,484) and single-organism process (10,031) were the most represented, and in molecular function binding (13,122) and catalytic activity (9,265) were most represented. KO annotation was used to classify 12,777 unigenes into five branches of the KEGG pathway (Figure 1B), including cellular processes (A), environmental information processing (B), genetic information processing (C), metabolism (D), and organismal systems (E).

### Identification of putative odorant receptors

Antennal transcriptome analysis of CLB samples identified 53 putative ORs (File S1). Among these, 11 sequences contained a full-length ORF, and five genes (AchiOR1, AchiOR24, AchiOR32, AchiOR43, and AchiOR44) contained seven-transmembrane domains (Table S2). We identified an OR gene (AchiOR1) with a high sequence homology with the conserved Orco gene family of other insect species and have designated it as AchiOrco. Phylogenetic analysis in previous studies has divided coleopteran species ORs apart from the Orco gene subfamily (which includes AchiOrco, MaltOrco, McarOrco, PstrOrco, TmolOrco, and AcorOrco), into multiple subgroups numbered 1–7 (Engsontia et al., 2008; Andersson et al., 2013, Figure 2). 52 putative OR sequences were classified into four subgroups (group 1–3 and 7), with 19 sequences assigned to group 1, 18 sequences assigned to group 2, 10 sequences assigned to group 3, and five sequences assigned to group 7, respectively. Group 7 was further divided into two subsets: group 7a and group 7b. The remaining three subgroups 4–6 contained only *T. castaneum* sequences. Furthermore, 6 sequences (AchiOR22, AchiOR23, AchiOR26, AchiOR32, AchiOR34, and AchiOR44) were clustered with high orthology to pheromone receptors from *M. caryae*.

**Figure 2:**
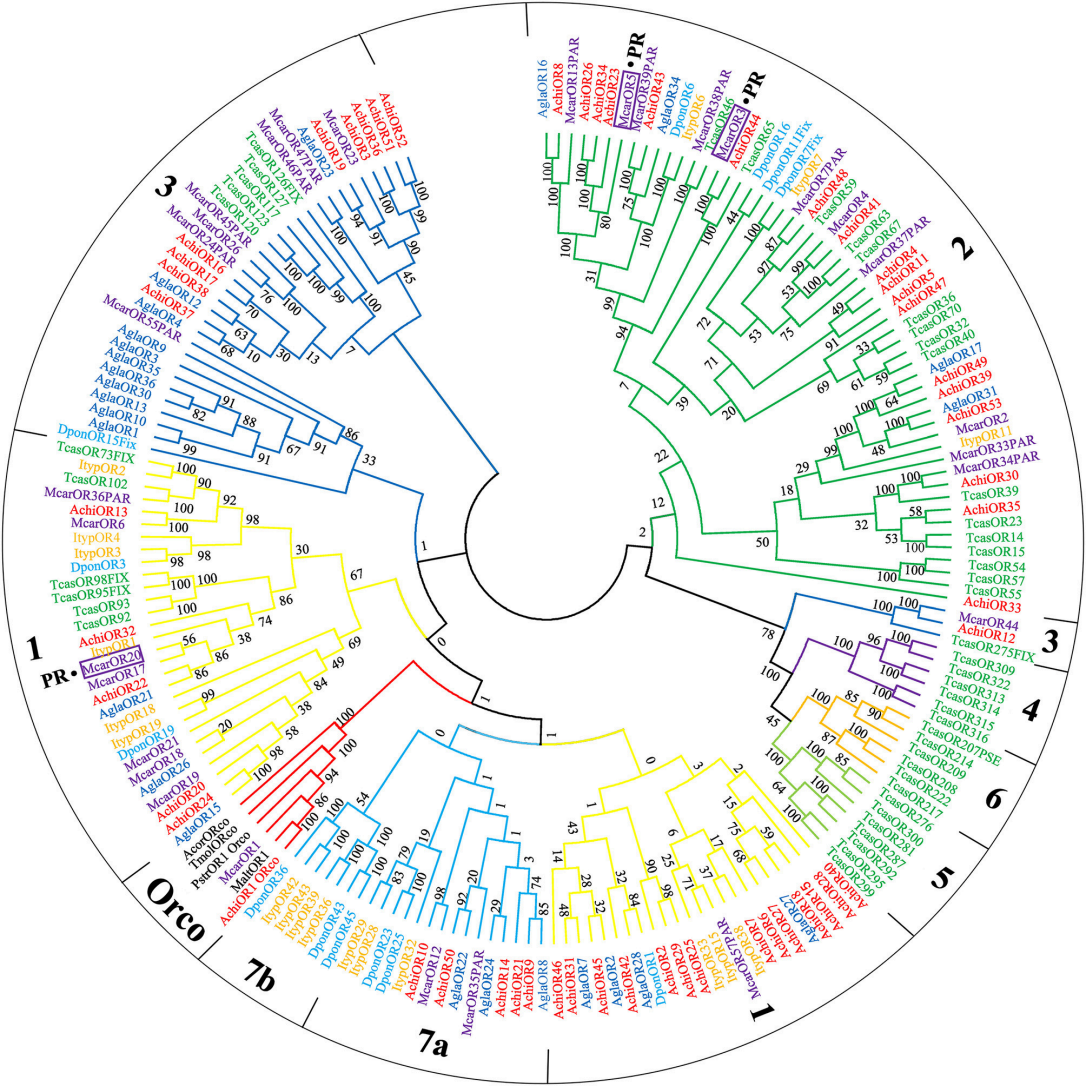
Phylogenetic tree of putative odorant receptor (OR) genes. The tree was constructed using MEGA6 with Neighbor-joining method. Achi: *A. chinensis*; Tcas: *T. castaneum*; Agla: *A. glabripennis*; Dpon: *D. ponderosae*; Ityp: *I. typographus*; Pstr: *P. striolata*; Acor: *A. corpulenta*; Malt: *M. alternates*; Tmol: *T. molitor*.

### Identification of putative gustatory receptors

**Figure 3:**
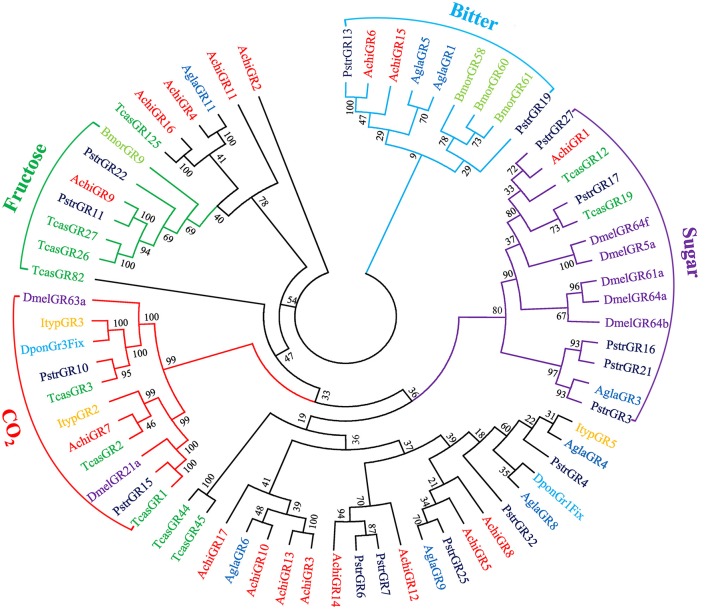
Phylogenetic tree of putative gustatory receptor (GR) genes. The tree was constructed using MEGA6 with Neighbor-joining method. Achi: *A. chinensis*; Tcas: *T. castaneum*; Agla: *A. glabripennis*; Dpon: *D. ponderosae*; Ityp: *I. typographus*; Pstr: *P. striolata*; Dmel: *D. melanogaster*; Bmor: *B. mori*.

Bioinformatic analysis identified 17 putative GRs in the CLB antennal transcriptome (File S1); four of which were full-length genes (Table S2). GR protein sequences from *A. chinensis* and seven additional insect species were used to construct a phylogenetic tree (Figure 3). In this tree, genes were classified into “sugar,” “fructose,” “bitter,” and “CO_2_” GR functions. AchiGR1 was highly homologous to known sugar receptors (Chyb et al., 2003; Dahanukar et al., 2007; Kent and Robertson, 2009), AchiGR9 was highly homologous with a novel fructose sugar receptor (Sato et al., 2011; Miyamoto and Amrein, 2014), AchiGR6 and AchiGR15 were highly homologous to known bitter receptors (Wanner and Robertson, 2008), and AchiGR7 was highly homologous to known carbon dioxide receptors (Kwon et al., 2007; Robertson and Kent, 2009).

### Identification of putative ionotropic receptors

**Figure 4:**
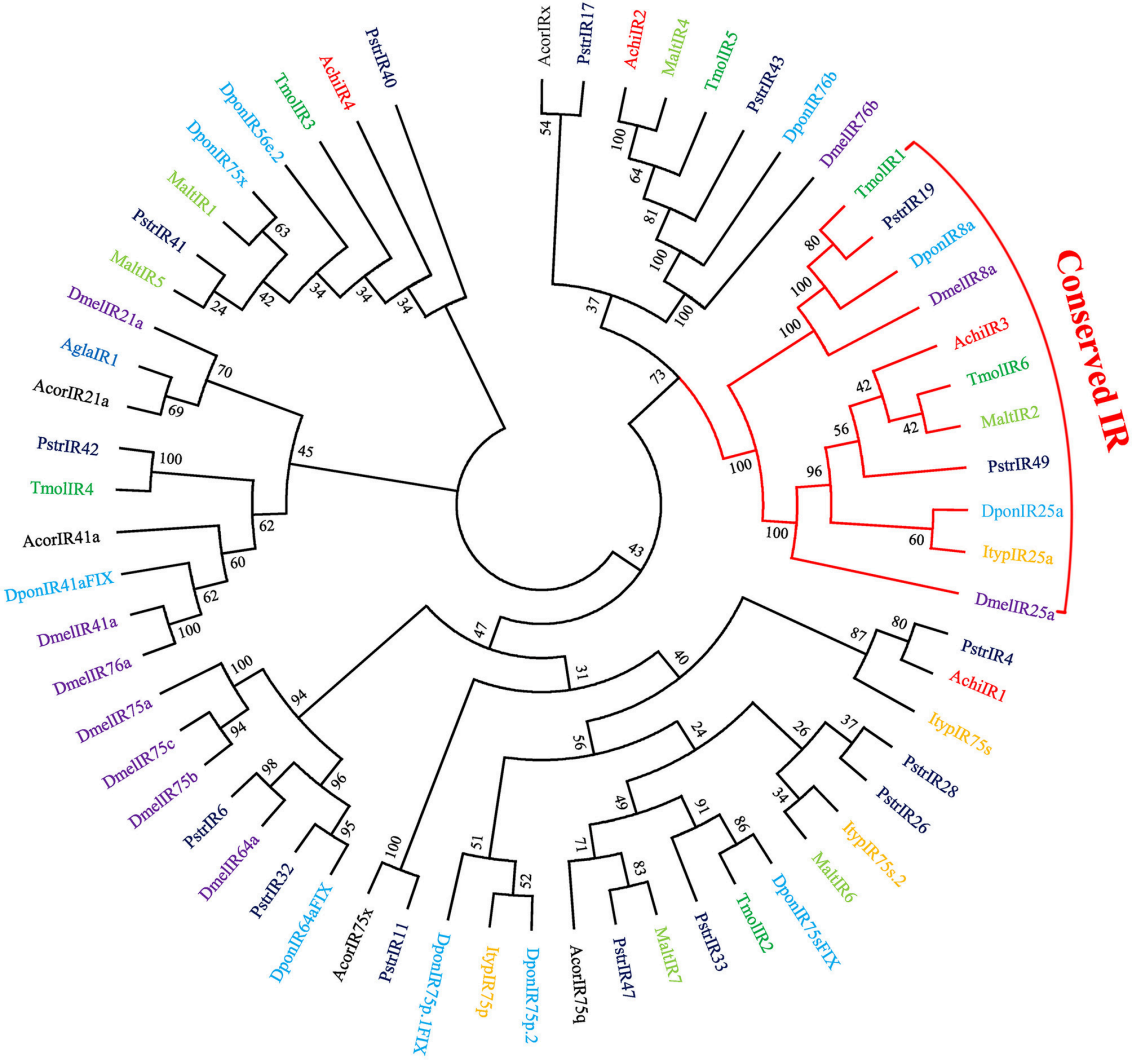
Phylogenetic tree of putative ionotropic receptor (GR) genes. The tree was constructed using MEGA6 with Neighbor-joining method. Achi: *A. chinensis*; Agla: *A. glabripennis*; Dpon: *D. ponderosae*; Ityp: *I. typographus*; Pstr: *P. striolata*; Acor: *A. corpulenta*; Malt: *M. alternates*; Tmol: *T. molitor*; Dmel: *D. melanogaster*.

Four putative IRs were identified in the combined antennal transcriptome (File S1). Among them, IR genes AchiIR2 and AchiIR3 had full-length ORFs, and the IR gene AchiIR4 was the only one without a transmembrane domain (Table S2). According to the phylogenetic analysis of IRs from eight species of coleopterans and *D. melanogaster* (Figure 4), IR genes can be classified into different subgroups. AchiIR2 clustered with DponIR76b and DmelIR76b at high percent identity, suggesting it belongs to the IR76b group. In addition, the phylogenetic tree classified AchiIR3 into the IR25a coreceptor subfamily.

### Tissue- and sex-specific expressions of putative chemosensory receptors

**Figure 5:**
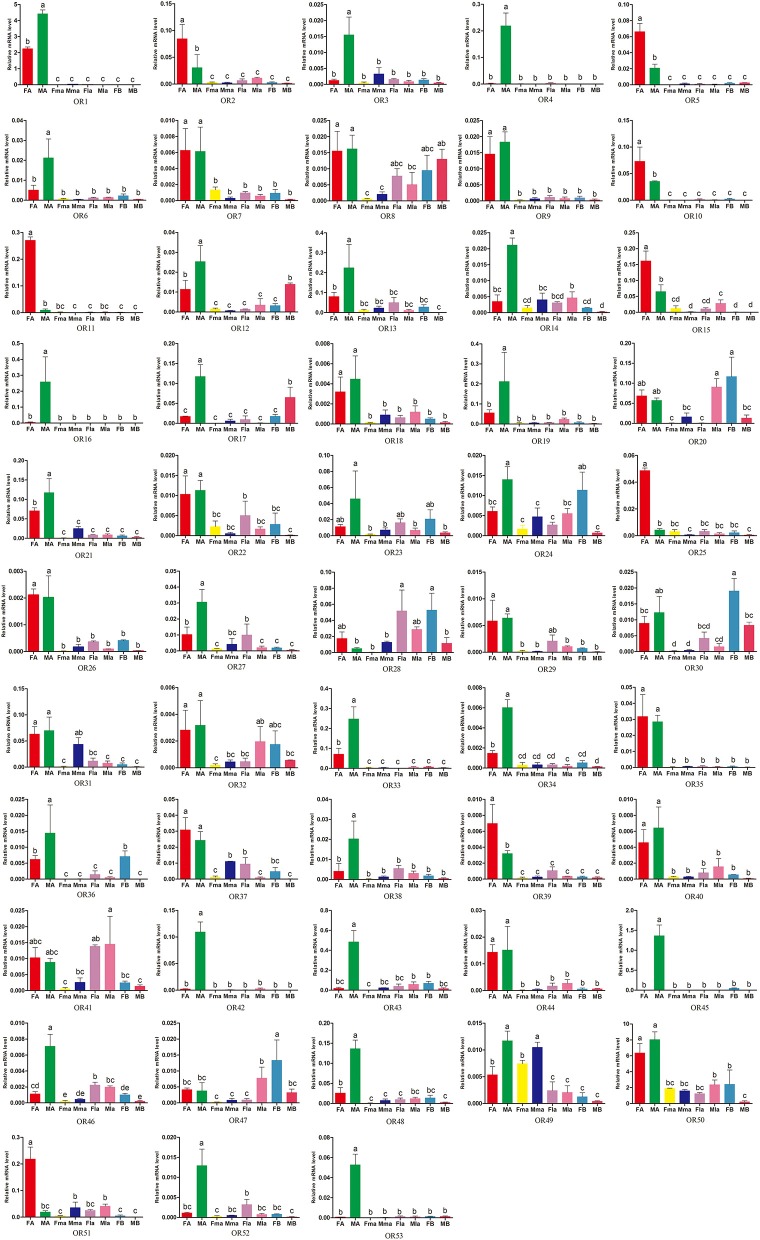
Expression levels of *A. chinensis* ORs in different tissues of female and male adults as measured by RT-qPCR. FA: female antennae, MA: male antennae, Fma: female maxillary palps, Mma: male maxillary palps, Fla: female labial palps, FB: female bodies (with antennae, maxillary palps and labial palps cut off), MB: male bodies (with antennae, maxillary palps and labial palps cut off). The bar represents standard error and the different small letters (a–d) above each bar indicate significant differences (*P* < 0.05).

Expression patterns of chemosensory receptors (53 ORs, 17 GRs, 4 IRs) in CLB antennae, maxillary palps, labial palps, and the remaining insect bodies of both sexes were determined using RT-qPCR. 41 putative OR genes were significantly expressed in the beetle antennae (Figure 5), of which antennal expression of 8 OR sequences (*AchiOR2, AchiOR5, AchiOR10-11, AchiOR15, AchiOR25, AchiOR39*, and *AchiOR51*) was significantly female-biased, antennal expression of 23 OR sequences (*AchiOR1, AchiOR3-4, AchiOR6, AchiOR12-14, AchiOR16-17, AchiOR19, AchiOR21, AchiOR27, AchiOR33-34, AchiOR36, AchiOR38, AchiOR42-43, AchiOR45-46, AchiOR48*, and *AchiOR52-53*) was significantly male-biased, and the remaining 10 OR sequences (*AchiOR7, AchiOR9, AchiOR18, AchiOR22, AchiOR26, AchiOR35, AchiOR37, AchiOR40, AchiOR44*, and *AchiOR50*) were expressed at the same or similar levels in both female and male antennae. In addition, *AchiOR49* was highly expressed in the maxillary palps. *AchiOR20, AchiOR28, AchiOR30*, and *AchiOR47* were expressed at a significantly higher level in female bodies. Finally, *AchiOR41* was highly expressed in the labial palps of both sexes.

**Figure 6:**
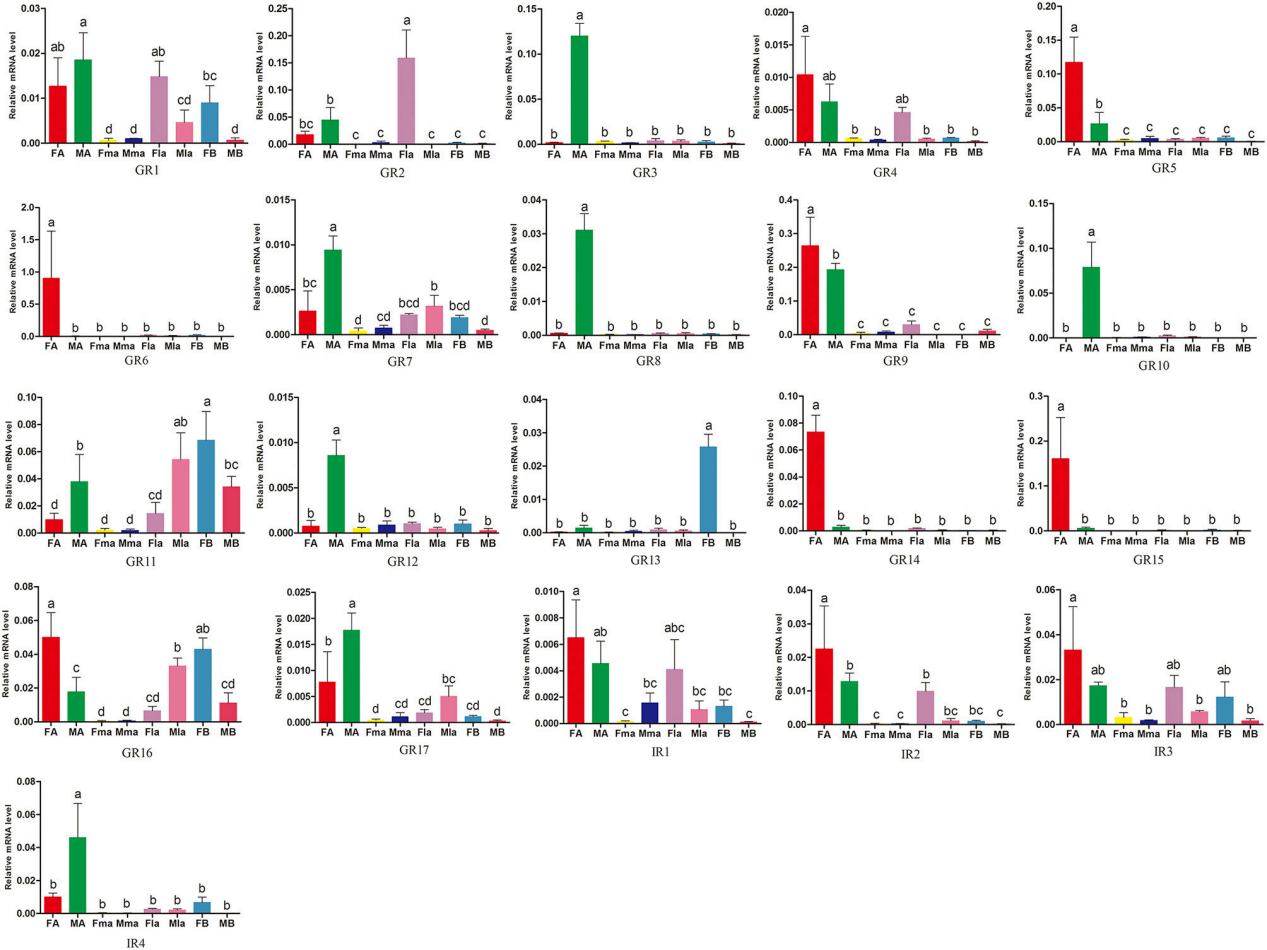
Expression levels of *A. chinensis* GRs and IRs in different tissues of female and male adults as measured by RT-qPCR. FA: female antennae, MA: male antennae, Fma: female maxillary palps, Mma: male maxillary palps, Fla: female labial palps, FB: female bodies (with antennae, maxillary palps and labial palps cut off), MB: male bodies (with antennae, maxillary palps and labial palps cut off). The bar represents standard error and the different small letters (a–d) above each bar indicate significant differences (*P* < 0.05).

11 of the 17 GR genes showed significantly higher expression in beetle antennae (Figure 6). Antennal expression of 5 GRs (*AchiGR5-6, AchiGR9*, and *AchiGR14-15*) was significantly female-biased, while antennal expression of the remaining 6 GRs (*AchiGR3, AchiGR7-8, AchiGR10, AchiGR12*, and *AchiGR17*) was significantly male-biased. *AchiGR2* expression in female labial palps was significantly higher than in any other tissues, while *AchiGR13* showed the highest expression in the female bodies. *AchiGR11* was highly expressed in male labial palps and female bodies. Among the four IRs identified, *AchiIR2* showed the highest expression in female antennae, whereas *AchiIR4* was mainly expressed in male antennae. In addition, *AchiIR1* and *AchiIR3* showed similar expression levels among all tested tissues (Figure 6).

## Discussion

Although Coleoptera is the largest insect order, the olfactory mechanisms of coleopterans at the molecular level are largely unknown. Furthermore, olfactory genes from Cerambycidae, an economically important coleopteran family, have only been partially identified in *M. alternatus* (Wang et al., 2014), *Batocera horsfieldi* (Li et al., 2014), *M. caryae* (Mitchell et al., 2012), *A. glabripennis* (Hu et al., 2016; Mitchell et al., 2017) and *A. chinensis* (Wang et al., 2017; and this paper).

In the transcriptome sets, a total of 44,938 unigenes were assembled from 89,331 transcripts, and 75.63% of these unigenes were longer than 500 bp, indicating the high depth and quality of the transcriptome sequences. The BLASTX homology analysis showed the best match with *T. castaneum* (55.6%), partly because a number of genes, including olfactory genes, were identified from genome data. GO and KO annotation exhibited some of the most represented subcategories: binding was the most abundant subcategory in the molecular function category, while signal transduction was the most abundant term in the environmental information processing pathway. The above unigenes may play vital roles in odorant binding and transduction activities in antennal chemosensory processes. CLB genes from the three multigene families of chemosensory receptors, including 53 ORs, 17 GRs, and 4 IRs, along with their expression patterns in different tissues of both sexes have now been identified through transcriptome analysis and RT-qPCR.

The 53 ORs identified in CLB were less than those identified in *T. castaneum* adult heads (111) (Engsontia et al., 2008) or *P. striolata* antennae and terminal abdomens (73) (Wu et al., 2016), but more than in *A. glabripennis* (37) (Hu et al., 2016), *A. planipennis* (2) (Mamidala et al., 2013), *A. corpulenta* (43) (Li et al., 2015), *M. alternatus* (9) (Wang et al., 2014), *Brontispa longissima* (48) (Bin et al., 2017), or *Rhyzopertha dominica* (6) (Diakite et al., 2016). According to the constructed OR phylogenetic tree (Figure 2), 52 putative OR sequences were distributed into four subgroups belonging to seven known coleopteran specific subgroups. In the present study, AchiOR1 was identified as AchiOrco due to the high level homology with the conserved Orco gene family, and clustered with other Orcos from *M. alternatus, M. caryae, P. striolata, T. molitor*, and *A. corpulenta*, probably attributed to the conserved nature of the chaperone OR. Interestingly, in the OR phylogenetic tree, six AchiOR genes, AchiOR22, AchiOR23, AchiOR26, AchiOR32, AchiOR34, and AchiOR44, were highly similar to three functionally characterized pheromone receptors (PRs), McarOR3, McarOR5, and McarOR20, from the cerambycid beetle *M. caryae*. Among them, AchiOR44 was orthologous to McarOR3, a receptor sensitive to the cerambycid pheromone (*S*)-2-methyl-1-butanol. AchiOR23, AchiOR26, and AchiOR34 formed a small clade around McarOR5, which is known to be sensitive to 2-phenylethanol, while AchiOR22 and AchiOR32 were clustered with McarOR20, a receptor of (2*S*,3*R*)-2,3-hexanediol and 3-hydroxyhexan-2-one. Mitchell et al. (2017) recently noted that the discovery of attractive volatile compounds could be expedited through further research on the expression of olfactory receptors. Due to their high level of sequence similarity to the three PRs, McarOR3, McarOR5, and McarOR20, these AchiORs may be associated with the detection of the above pheromones or other behaviorally active compounds. The discovery of new attractive substances for CLB is necessary for pest management as the currently known attractants have yet to be developed into a commercially viable attractive lure.

Previous research has revealed that most insect OR expression is localized in the antennae (Vosshall et al., 1999; Wang et al., 2015). In the current study, 41 ORs showed an antenna-specific expression profile. Of these, the 23 ORs with male-biased expression may play a significant role in sensing female-produced sex pheromones and female-acquired host-derived sexual attractants, while the 8 ORs with female-biased expression may be involved in some female specific behaviors such as oviposition site seeking, and 10 ORs, which were not biased toward either sex, may be associated with an aggregation pheromone or the detection of plant volatiles. Notably, AchiOR34 showed a clear male-biased expression profile and was clustered with pheromone receptors of *M. caryae* on the phylogenetic tree (Figure 2), strongly suggesting that it might be a pheromone receptor for sensing a female-produced sex pheromone in CLB. The ORs with high maxillary or labial palp expression may be involved in host selection for both sexes and oviposition site selection for females. A few ORs highly expressed in non-olfactory tissues is consistent with what has been reported in other insects (Li et al., 2015; Zhang et al., 2016; Zhao et al., 2016).

Of the 17 putative GRs belonging to four function groups, AchiGR1 is a probable sugar receptor, AchiGR9 shared a high similarity with the fructose receptor family members that respond to D-fructose such as DmelGR43a and BmorGR9 (Sato et al., 2011; Miyamoto and Amrein, 2014), AchiGR6 and AchiGR15 showed a high degree of similarity to the bitter receptor family, and AchiGR7 may be involved in detecting CO_2_. Similarly to ORs, most GRs were prominently expressed in antennae, likely because all GRs were identified from antennal transcriptome rather than the complete genome. Several other GRs that were highly expressed in labial palps or other gustatory organs are likely involved in detecting soluble stimulants and feeding behaviors. Only 4 IRs were identified in CLB, less than that in *P. striolata* (49) (Wu et al., 2016) or *B. longissima* (19) (Bin et al., 2017), but similar to the number identified from long-horned beetles, *A. glabripennis* (4) (Hu et al., 2016) or *M. alternates* (7) (Wang et al., 2014). In the IR phylogenetic tree, AchiIR2 clustered with IR76b orthologs, while AchiIR3 clustered with coreceptor IR25a orthologs. Compared to ORs and GRs, IRs are involved in regulating sensory transduction of olfaction and gustation, and are expressed in both olfactory and gustatory organs (Croset et al., 2010; van Giesen and Garrity, 2017). Two of the four identified IRs showed markedly antennae-biased expression while the remaining two IRs were widely expressed in all the tested tissues.

As we were finalizing our present manuscript for submission, an independent and complementary work on CLB was published online by Wang et al. (2017) that focused on olfactory-binding-protein gene families (OBPs and CSPs) rather than chemosensory receptor superfamilies. A total of 44 ORs, 19 GRs, and 23 IRs were identified by Wang et al. (2017), while 53 ORs, 17 GRs, and 4 IRs were identified in our current study. Five of our 17 AchiGRs had 100% identity with a counterpart and one AchiIR out of our four AchiIRs matched 100% with a corresponding IR reported in Wang et al. (2017). Only nine AchiORs presented 100% identity with a corresponding AchiORs. Notably, our AchiOR1 in our study, which clustered well with MaltOR01 and McarOR01, showed 100% identity with AchiOR35 in Wang et al. (2017), and both were defined as the conserved Orco gene. Our phylogenetic trees included sequences from *M. caryae* and *A. glabripennis* rather than those from *Bombyx mori* (Wang et al., 2017), and showed six of our AchiORs (AchiOR22, AchiOR23, AchiOR26, AchiOR32, AchiOR34, and AchiOR44) clustered well with three of the PR genes in *M. caryae*. An overview comparison of the receptor gene sequences identified in our study and Wang et al. (2017) using NCBI protein-protein BLASTP 2.6.0+ indicated that only 40 of our 74 identified receptors matched their recently published receptor genes with >90% amino acid identity (Table S3). We attribute our different identifications to our greater sample size, differences in collection sites and host plants, or other unseen reasons.

In the present study, ORs genes from the congener *A. glabripennis* were used to generate the neighbor-joining phylogenetic trees, surprisingly, these genes were not included in Wang et al. (2017) paper. An comparison of the 53 AchiORs identified in our study and 37 AglaORs in Hu et al. (2016) using NCBI protein-protein BLASTP 2.6.0+ indicated that at least 15 of our putative AchiORs showed high amino acid identities (up to 80%) with AglaORs (Hu et al., 2016, Table S4). Among them, AchiOR43 and AchiOR53 both had 100% identity with their corresponding genes AglaOR29 and AglaOR31 and the other four AchiORs (AchiOR8, AchiOR14, AchiOR19, and AchiOR49) had at least 95% identity with corresponding AglaORs. Closely related cerambycid species often share pheromones or pheromone motifs (Millar and Hanks, 2017). As the congeners CLB and *A. glabripennis* are previously known to both use 4-(*n*-heptyloxy)butanol as part of their pheromone systems (Hansen et al., 2015), the high level of homology between the AchiORs and AglaORs suggests that one or several of these may be pheromone receptor(s) tuned to 4-(*n*-heptyloxy)butanol. Further research on the functional characteristics of these receptors is surely needed.

Additionally, we conducted a further similarity analysis on the ORs to compare our 53 AchiORs with the 132 AglaORs sequences from the genome of *A. glabripennis* reported by Mitchell et al. (2017) (Table S5). Interestingly, all of the 53 AchiORs (except two ORs, AchiOR18, and AchiOR27) shared at least a 73% amino acid identity with AglaORs from the *A. glabripennis* genome, and 28 AchiORs had at least 95% identity with corresponding AglaORs. More importantly, our AchiOR1 (AchiOrco) was incredibly similar to AglaOR1/Orco, with 99.6% identity. This result further verified the attribute of AchiOR1 as a conserved Orco gene.

## Conclusion

In order to better understand the olfactory system molecular mechanisms of CLB, a polyphagous long-horned beetle that infests a wide range of broadleaved trees across many countries, we generated its antennal transcriptome. We then identified 74 putative receptor genes from the chemosensory receptor gene families, including 53 ORs, 17 GRs, and 4 IRs through bioinformatic analysis. RT-qPCR generated expression profiles of these chemosensory receptors demonstrated that most were prominently expressed in antennae, especially in male antennae, indicating that they may play a critical role in sensing sex pheromones. Functional characterization of putative pheromone receptors such as AchiOR34 in order to explore their binding capacity to known ceramycid pheromones, particularly pheromones of both *A. glabripennis* and *A. chinensis*, is a highly attractive future research objective. Our discovery of these chemosensory receptors may lead to a new perspective for controlling these economically important pest insects.

## Author contributions

L-WZ, LS, and Y-NZ: Conceived and designed the experiments; LS, J-DD, J-LQ, KK, and Y-NZ: Performed the experiments; LS, L-WZ, X-QZ, KK, Y-NZ, CC, and Y-PT: Analyzed the data; L-WZ and Y-NZ: Contributed reagents, materials, analysis tools; LS, L-WZ, KK, Y-NZ, LH, TX, and Q-HZ: Wrote the paper.

### Conflict of interest statement

Author Qing-He Zhang is an employee at Sterling International, Inc. (SII), but not a SII shareholder or officer, and thus has no financial conflict of interest as it related to this work. The other authors declare that the research was conducted in the absence of any commercial or financial relationships that could be construed as a potential conflict of interest.

## Supplementary material

Figure S1 Distribution of unigenes and transcript lengths in the *Anoplophora chinensis* transcriptome assembly.

Figure S2 Species distribution of homology search with the *Anoplophora chinensis* unigenes against the Nr database.

Table S1 Primes used for quantitative real time-PCR.

Table S2 Blastx matches for the putative chemosensory receptor genes of *Anoplophora chinensis*.

Table S3 Comparative overview of *Anoplophora chinensis* chemosensory receptor genes idenetified in this study and in Wang et al. (2017) study.

Table S4 Comparative overview of *Anoplophora chinensis* chemosensory receptor genes identified in this study and *Anoplophora glabripennis* chemosensory receptor genes reported in Hu et al. (2016) study.

Table S5 Comparative overview of *Anoplophora chinensis* odorant receptor genes identified in this study and *Anoplophora glabripennis* OR genes reported in Mitchell et al. (2017) study.

File S1 The amino acid sequences of *Anoplophora chinensis* putative chemosensory receptor genes.
