# Supplementary figures and images for: Identification and Expression Patterns of Anoplophora chinensis (Forster) Chemosensory Receptor Genes from the Antennal Transcriptome
Source: Front Physiol. 2018 Feb 13;9:90. doi: 10.3389/fphys.2018.00090 (PMC5819563; doi:10.3389/fphys.2018.00090)

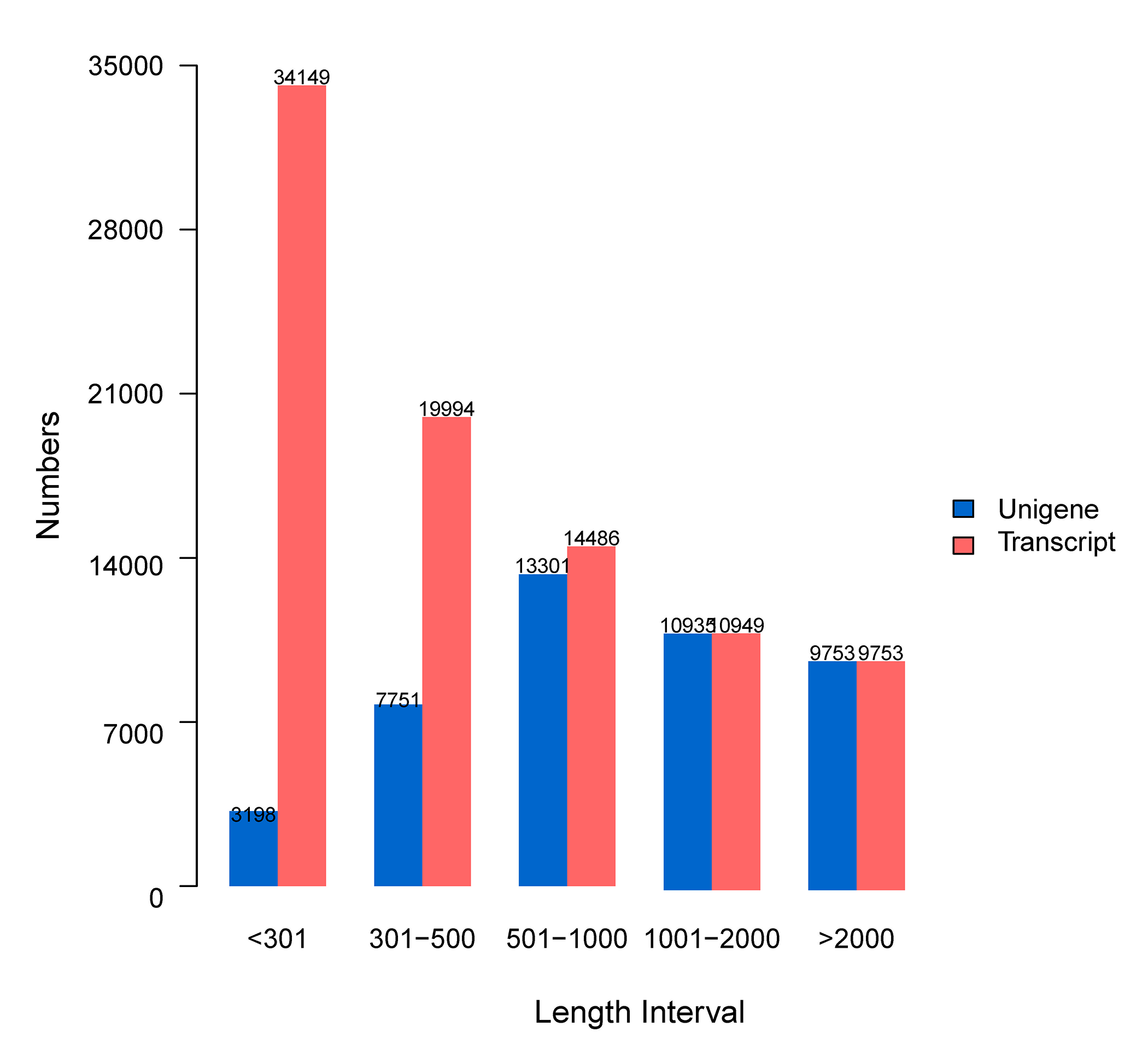

Supplement: Figure S1 — Distribution of unigenes and transcript lengths in the Anoplophora chinensis transcriptome assembly. [file Image1.TIF]

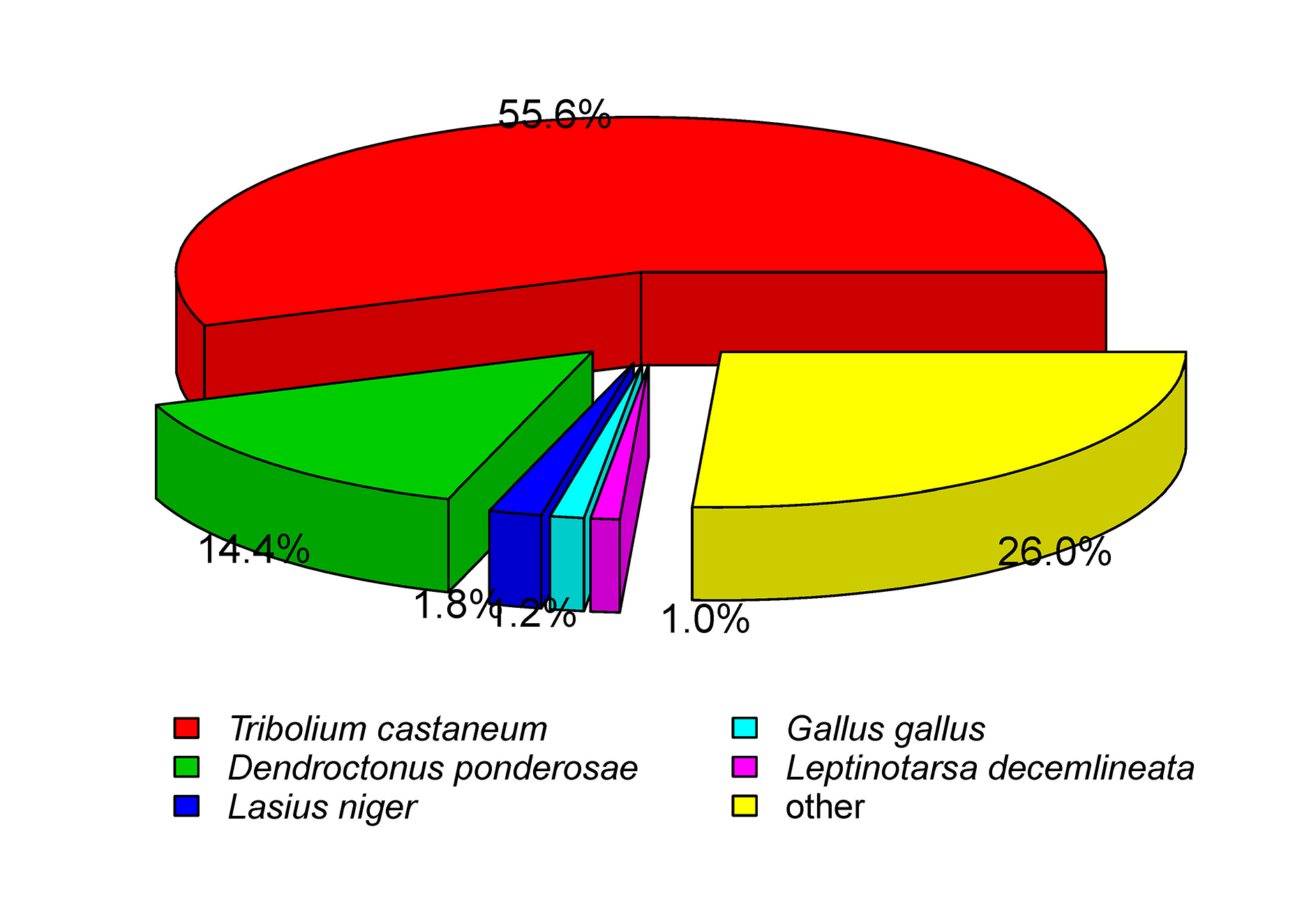

Supplement: Figure S2 — Species distribution of homology search with the Anoplophora chinensis unigenes against the Nr database. [file Image2.TIF]
