# Supplementary material for: Identification and Expression Patterns of Anoplophora chinensis (Forster) Chemosensory Receptor Genes from the Antennal Transcriptome
Source: Front Physiol. 2018 Feb 13;9:90. doi: 10.3389/fphys.2018.00090 (PMC5819563; doi:10.3389/fphys.2018.00090)
Supplement: Table S1 — Primes used for quantitative real time-PCR. [file Table1.DOCX]

**Table S1** Primes used for quantitative real time-PCR.

| **Gene name** | **Forward primer** | **Reverse primer** | **Gene accession number** |
| --- | --- | --- | --- |
| *Odorant receptors (ORs)* | | |  |
| OR1(Orco) | CCTACCAAGCTACCAAAA | CCATGACAGAAGAACTCTC | MG766143 |
| OR2 | AGGAGCTTGCATTGTTAA | TGTCGTATTCTAACCATGG | MG766144 |
| OR3 | GACCTATAGTACGTCTGATC | TGTCGTACCATCTATTGAA | MG766145 |
| OR4 | CCGAATTAGTACGAGATACA | GGGATAATTAACAGCTTAAACA | MG766146 |
| OR5 | CGAGTAGTACAATCTCAGAA | CAGACCTCCATATACTCATTA | MG766147 |
| OR6 | CGTAGTAATGTGCGTTTG | TTCGCTCATGTATATGTCA | MG766148 |
| OR7 | TTCGGCATTATATTACATGTC | GCTGCAACTGAAACTATC | MG766149 |
| OR8 | CTTCCGGAGAACTACAAG | GCCACGTATAAACATGATG | MG766150 |
| OR9 | GCGTTCTCTATGCTATTAAG | GCAGCATCATTTGTTTTAC | MG766151 |
| OR10 | GTCTCTAACCTTTGCATAAC | CCAACTTGAATACATTCTGAA | MG766152 |
| OR11 | CTCAGTCAATTGCTTATCTC | GACCAAATTTTAATTTATTCCTCA | MG766153 |
| OR12 | GCGTTTGCCATTATACTC | CTGACAAGTGGACCATAA | MG766154 |
| OR13 | GGACAGATGATATTAGATGAGA | CTACGCCTATAGAAGCTAC | MG766155 |
| OR14 | CCGCAATACTCATCTTTATC | GTAGCTGATGAGGTAGTG | MG766156 |
| OR15 | GAAGGAACAACTGAAGAATA | GCTTGATGAATTTCTGTCA | MG766157 |
| OR16 | ACGACTTTGACAGATACG | TGGCACATGTAGTAATTCA | MG766158 |
| OR17 | TGCGTATAAATGGTACTTATC | AGTCCTGTTGTTAATAATATAATAG | MG766159 |
| OR18 | GACACATTTCTGCACATG | CGTGTATTCATCAAACTTGTC | MG766160 |
| OR19 | CAGGAATGTTCCAATATACTG | CACCTATCATTAGTGTTGTAG | MG766161 |
| OR20 | GGTGTTTGGATGTATTGG | CGTACCATTTTGTGTCATA | MG766162 |
| OR21 | GCTAGTCTATTACTGGTACG | CTCTGAATCACAAGTAGCA | MG766163 |
| OR22 | GGCTCTGTTAGTTCATTTC | CGTCATATAGGTCAGTACC | MG766164 |
| OR23 | CACGTTGATTGGATGTTAC | CGAGTTTAGGTAGAGTTTCA | MG766165 |
| OR24 | CGACGTGCAAATTATGAG | CAACGTGAGATAGGAATATG | MG766166 |
| OR25 | TACGGCAAATACTCCAAA | TCAGCTATGCCAATACTC | MG766167 |
| OR26 | ACTGCAAGTGAAGAGTTA | CTGTCGCCTTAAGAGTTA | MG766168 |
| OR27 | CCATGGTTATGGAATATTAAGC | GCACAATAATTCTCGGGAA | MG766169 |
| OR28 | GCAGTACCATTCATCTAATG | CTGTGTTGAAGAAATGTCA | MG766170 |
| OR29 | ACACGAACAAGTAGTCAA | CCTCAAATGGTATCCACATA | MG766171 |
| OR30 | ACAGCATTAACAGAAAGGA | CCACGAAGGGATAGATTC | MG766172 |
| OR31 | CAACCAAGTGCGAATATG | CGTCTTGACACAATTTAATAAAG | MG766173 |
| OR32 | CCTTGGCTATATCTTTATTTGG | GCGTAAAAGTCCTTTGGA | MG766174 |
| OR33 | CAGCATTATTGCGATCTG | GTGACAAATTATCCATCTCAA | MG766175 |
| OR34 | ACTGCAAGTGAAGAGTTA | CTGTCGCCTTAAGAGTTA | MG766176 |
| OR35 | GGTCCTGAGTGTTTTACA | CTGCATATGGAATCTTCTTAC | MG766177 |
| OR36 | CCATGTGCATTCAAATGTA | TGCTGAAGATGGATTCTC | MG766178 |
| OR37 | GGTTCAAGTTAGACTGATTATG | GTACCATATGCACTCAGAA | MG766179 |
| OR38 | CAGCAACAGCAGACATTA | GCCATCAGTAGAACAGTTC | MG766180 |
| OR39 | CCTTGGATAGGCTGTAAC | GTCGTTCGTAGTATTGAGA | MG766181 |
| OR40 | CCACGGACTAACAAAATTC | GTACGAGTAAGAGCACTG | MG766182 |
| OR41 | CGCAATGCCTTCAATATC | GGACGACGTTATCAATAAAG | MG766183 |
| OR42 | GTTGCTATAACAGACGTAAC | GCGGATATTGTTTCTTTTGA | MG766184 |
| OR43 | CCACTTTATTTTCTTCATACTTG | GGCATCCAACTGTAATATG | MG766185 |
| OR44 | TCGACACAGGATATAACG | GTGGTTTCAATCGGTAAC | MG766186 |
| OR45 | CGCCTGAAGGAATATCTAA | GCGTGATGAAACTTGAAA | MG766187 |
| OR46 | CCCTGGAAATACCGAATA | AGGTCATTGCTCATAGTATA | MG766188 |
| OR47 | ATCGGACATCATCTACAAG | CGGTATGGTAAACGGTAC | MG766189 |
| OR48 | GCATCGACCTATTGACTA | TCGTCGTAGTGAATAATACA | MG766190 |
| OR49 | CCGAAAGGATGTATAAATTAATG | AGAGGCTTAGTATAGTCGTA | MG766191 |
| OR50 | CAACGAGTTTGTGAAGAG | ACGGTATGGGAAATATAGC | MG766192 |
| OR51 | CCAAGCAATGATGTATGC | GTTCGTTCCAGTTATTGAAG | MG766193 |
| OR52 | GCATCAGTACAGAAATCAATA | CCAGTTGTTGAAGTATACG | MG766194 |
| OR53 | CGTACAGCATCATAGAGA | GCATGCAAATTGAAAAGAA | MG766195 |
| *Gustatory receptors (GRs)* | | |  |
| GR1 | GCAGAAATAGATTGTGAGAG | CTCCAGCTATACGTAACAA | MG766196 |
| GR2 | CGTCTTTAACTCACTAGGTA | CTGACACTATACGGAACC | MG766197 |
| GR3 | GCTGTAAAAGAGGCAGAA | GGTTGTAGTAGTTCCAAGTA | MG766198 |
| GR4 | CCGTATCTGAGGATCAAG | CAGGTAAATGTTGACTATGC | MG766199 |
| GR5 | CGGCAAGAAAATCATCAA | CAGGTTTCCATTGTTCAG | MG766200 |
| GR6 | ACTCCTAGTTATCTATTCCAAC | CACTGAAATCTTTTACTTTGAAG | MG766201 |
| GR7 | GTCCAACTGTCTTTGATG | GAGGTAGATAGCAATGGTA | MG766202 |
| GR8 | GAAGCAAATTGGCAAAATC | GTTGGTAACACTGACTGA | MG766203 |
| GR9 | GCTCATATTGGAAGGCTA | GTTGCATGGAGAATATTATCA | MG766204 |
| GR10 | GGTAGACGACTTCATAGAC | CACTGACTGAACCAGAAA | MG766205 |
| GR11 | TCACGTACTATCCACTTTG | CCACCAGTAACAGATACAA | MG766206 |
| GR12 | TCTGCTAGTAGTTTTGGC | GCACTTAATCTAGGATTTCTG | MG766207 |
| GR13 | CGACTGGGCTATAAAAGA | CTGGTTTGTTGTTGACAA | MG766208 |
| GR14 | TGGTGCGAGTATACGATA | GCAAGGAAAAGCTGATTAC | MG766209 |
| GR15 | GCGACATATTTTGGATTCC | CTTGGTTAAACAATTCAGTGTA | MG766210 |
| GR16 | GAGAGAATTAAACAAGACCTTC | CCACCATCTCATCAACTAA | MG766211 |
| GR17 | GTAGCCCATTCATTTGCA | TGCCTTGAGTACGTTATC | MG766212 |
| *Ionotropic receptors (IRs)* | | |  |
| IR1 | CAAGGTGAACTATGTGCTA | GTCTGCTTCATTTCTAGTAAG | MG766213 |
| IR2 | GTGGTCTTGATGAAGAGA | TCTGGCTTGTATTAGTATCA | MG766214 |
| IR3 | GCTCTTTGGTTTCATCATTA | CTGGATCTTGTACTGCTTA | MG766215 |
| IR4 | AGAGCAGGACTTATTGAAA | CACTGATTTTGGACATAACA | MG766216 |
| *Endogenous reference genes* | | |  |
| GAPDH | GGAGTATTCACAACCATTG | GCATCAAGATTAACACCG |  |
| Actin | CGACTTGACTGACTATCTC | GGTAGTTCGTAGCTCTTC |  |
