# Supplementary material for: Identification and Expression Patterns of Anoplophora chinensis (Forster) Chemosensory Receptor Genes from the Antennal Transcriptome
Source: Front Physiol. 2018 Feb 13;9:90. doi: 10.3389/fphys.2018.00090 (PMC5819563; doi:10.3389/fphys.2018.00090)
Supplement: Table S2 — Blastx matches for the putative chemosensory receptor genes of Anoplophora chinensis. [file Table2.docx]

**Table S2** Blastx matches for the putative chemosensory receptor genes of *Anoplophora chinensis*.

| **Gene**  **Name** | **ORF Length(bp)** | **Complete**  **ORF** | **FPKM**  **value** | **TM**  **Number** |  | **Best Blastx Match** | | | |
| --- | --- | --- | --- | --- | --- | --- | --- | --- | --- |
|  |  |  |  |  | **Name** | **Species** | **Acc. number** | **E value** | **Identity(%)** |
| OR1 (Orco) | 1434 | YES | 98.3 | 7 | olfactory receptor 1 | *Monochamus alternatus* | AIX97092.1 | 0.0 | 97% |
| OR2 | 384 | NO | 2.32 | 0 | odorant receptor 10 | *Anopheles sinensis* | KFB39838.1 | 2e-17 | 43% |
| OR3 | 585 | NO | 2.64 | 1 | odorant receptor OR5 | *Colaphellus bowringi* | ALR72550.1 | 2e-28 | 39% |
| OR4 | 582 | NO | 1.21 | 3 | olfactory receptor 5 | *Monochamus alternatus* | AIX97096.1 | 9e-55 | 85% |
| OR5 | 288 | NO | 0.85 | 0 | odorant receptor OR9 | *Colaphellus bowringi* | ALR72554.1 | 1e-18 | 47% |
| OR6 | 216 | NO | 1.69 | 1 | odorant receptor OR3 | *Colaphellus bowringi* | ALR72548.1 | 3e-11 | 46% |
| OR7 | 444 | NO | 1.16 | 1 | odorant receptor OR40 | *Colaphellus bowringi* | ALR72583.1 | 2e-10 | 32% |
| OR8 | 666 | NO | 1.18 | 2 | odorant receptor OR36 | *Colaphellus bowringi* | ALR72579.1 | 3e-109 | 73% |
| OR9 | 324 | NO | 2.91 | 2 | odorant receptor 8 | *Dendroctonus ponderosae* | AKK25155.1 | 1e-13 | 43% |
| OR10 | 1275 | YES | 0.43 | 6 | odorant receptor 60 | *Tribolium castaneum* | EEZ99415.2 | 2e-17 | 29% |
| OR11 | 930 | NO | 1.1 | 5 | odorant receptor 8 | *Pyrrhalta aenescens* | APC94315.1 | 3e-26 | 28% |
| OR12 | 1209 | YES | 2.09 | 6 | odorant receptor 18 | *Pyrrhalta maculicollis* | APC94230.1 | 2e-98 | 41% |
| OR13 | 300 | NO | 1.77 | 2 | odorant receptor 102 | *Tribolium castaneum* | EEZ97750.2 | 3e-21 | 49% |
| OR14 | 438 | NO | 0.92 | 0 | odorant receptor 26 | *Tribolium castaneum* | EEZ99239.1 | 0.003 | 28% |
| OR15 | 423 | NO | 1.36 | 2 | odorant receptor 18 | *Pyrrhalta maculicollis* | APC94230.1 | 1e-32 | 54% |
| OR16 | 876 | NO | 1.49 | 6 | odorant receptor OR40 | *Colaphellus bowringi* | ALR72583.1 | 6e-28 | 30% |
| OR17 | 195 | NO | 1.85 | 0 | olfactory receptor 9 | *Monochamus alternatus* | AIX97100.1 | 8e-11 | 44% |
| OR18 | 147 | NO | 5.6 | 0 | odorant receptor OR32 | *Colaphellus bowringi* | ALR72575.1 | 1e-06 | 55% |
| OR19 | 1131 | NO | 2.01 | 4 | odorant receptor OR37 | *Colaphellus bowringi* | ALR72580.1 | 1e-58 | 36% |
| OR20 | 345 | NO | 1.55 | 2 | odorant receptor OR6 | *Colaphellus bowringi* | ALR72551.1 | 1e-41 | 56% |
| OR21 | 369 | NO | 0.98 | 0 | odorant receptor 15 | *Dendroctonus ponderosae* | AKK25156.1 | 4e-10 | 34% |
| OR22 | 1155 | YES | 2.8 | 6 | odorant receptor OR24 | *Colaphellus bowringi* | ALR72568.1 | 9e-130 | 46% |
| OR23 | 852 | NO | 1.25 | 4 | odorant receptor OR36 | *Colaphellus bowringi* | ALR72579.1 | 2e-92 | 48% |
| OR24 | 1158 | YES | 1.93 | 7 | odorant receptor OR6 | *Colaphellus bowringi* | ALR72551.1 | 7e-102 | 42% |
| OR25 | 366 | NO | 3.15 | 2 | odorant receptor 2 | *Anopheles stephensi* | ACH69148.1 | 2e-05 | 30% |
| OR26 | 438 | NO | 0 | 2 | odorant receptor OR36 | *Colaphellus bowringi* | ALR72579.1 | 7e-46 | 53% |
| OR27 | 168 | NO | 0.15 | 0 | odorant receptor 315 | *Tribolium castaneum* | EFA02958.1 | 6e-04 | 29% |
| OR28 | 291 | NO | 4.9 | 0 | odorant receptor 49b | *Drosophila obscura* | XP_022220330.1 | 3e-15 | 38% |
| OR29 | 939 | NO | 3.54 | 4 | odorant receptor 2 | *Aedes aegypti* | NP_001345252.1 | 1e-10 | 26% |
| OR30 | 1098 | NO | 1.5 | 5 | odorant receptor 3 | *Pyrrhalta aenescens* | APC94308.1 | 2e-110 | 45% |
| OR31 | 648 | NO | 1.43 | 4 | odorant receptor OR36 | *Colaphellus bowringi* | ALR72579.1 | 3e-53 | 41% |
| OR32 | 1155 | YES | 2.73 | 7 | odorant receptor OR17 | *Colaphellus bowringi* | ALR72562.1 | 1e-109 | 44% |
| OR33 | 1074 | NO | 1.72 | 5 | odorant receptor 4 | *Pyrrhalta aenescens* | APC94309.1 | 2e-74 | 37% |
| OR34 | 438 | NO | 0.72 | 2 | odorant receptor OR36 | Colaphellus bowringi | ALR72579.1 | 7e-46 | 53% |
| OR35 | 339 | NO | 1.22 | 1 | olfactory receptor OR11 | *Tenebrio molitor* | AJO62230.1 | 8e-25 | 53% |
| OR36 | 600 | NO | 2.32 | 1 | odorant receptor OR5 | *Colaphellus bowringi* | ALR72550.1 | 3e-37 | 40% |
| OR37 | 459 | NO | 2.54 | 2 | odorant receptor OR40 | *Colaphellus bowringi* | ALR72583.1 | 2e-22 | 33% |
| OR38 | 222 | NO | 1.23 | 0 | olfactory receptor 9 | *Monochamus alternatus* | AIX97100.1 | 2e-14 | 44% |
| OR39 | 627 | NO | 1.04 | 3 | odorant receptor OR26 | *Colaphellus bowringi* | ALR72569.1 | 2e-54 | 49% |
| OR40 | 324 | NO | 1.84 | 2 | odorant receptor 60 | *Tribolium castaneum* | EEZ99415.2 | 1e-21 | 42% |
| OR41 | 1149 | YES | 2.51 | 6 | olfactory receptor OR16 | *Tenebrio molitor* | AJO62235.1 | 3e-96 | 43% |
| OR42 | 504 | NO | 2.72 | 2 | odorant receptor OR28 | *Colaphellus bowringi* | ALR72571.1 | 3e-15 | 33% |
| OR43 | 1254 | YES | 1.24 | 7 | odorant receptor 26 | *Pyrrhalta aenescens* | APC94330.1 | 0.0 | 64% |
| OR44 | 1308 | YES | 3.98 | 7 | olfactory receptor 14 | *Tribolium castaneum* | CAM84012.1 | 4e-100 | 42% |
| OR45 | 468 | NO | 1.42 | 2 | putative odorant receptor OR53 | *Athetis lepigone* | AOE48058.1 | 0.30 | 33% |
| OR46 | 450 | NO | 0.96 | 3 | odorant receptor OR20 | *Colaphellus bowringi* | ALR72565.1 | 7e-22 | 34% |
| OR47 | 1140 | NO | 5.27 | 5 | odorant receptor OR3 | *Colaphellus bowringi* | ALR72548.1 | 9e-100 | 50% |
| OR48 | 1170 | YES | 1.22 | 6 | odorant receptor 8 | *Pyrrhalta aenescens* | APC94315.1 | 2e-109 | 44% |
| OR49 | 951 | NO | 0.94 | 5 | odorant receptor OR26 | *Colaphellus bowringi* | ALR72569.1 | 9e-83 | 44% |
| OR50 | 717 | NO | 1.04 | 4 | odorant receptor 28 | *Anomala corpulenta* | AKC58563.1 | 8e-06 | 27% |
| OR51 | 1143 | YES | 1.98 | 4 | odorant receptor OR28 | *Colaphellus bowringi* | ALR72571.1 | 9e-46 | 31% |
| OR52 | 1128 | NO | 2.28 | 5 | odorant receptor OR28 | *Colaphellus bowringi* | ALR72571.1 | 2e-44 | 30% |
| OR53 | 312 | NO | 0.87 | 2 | odorant receptor OR12 | *Colaphellus bowringi* | ALR72557.1 | 3e-31 | 51% |
| GR1 | 303 | NO | 2.17 | 2 | Putative gustatory receptor 64f | *Acromyrmex echinatior* | EGI60659.1 | 1e-22 | 47% |
| GR2 | 741 | NO | 1.01 | 4 | gustatory receptor 2 | *Colaphellus bowringi* | ALR72528.1 | 5e-26 | 46% |
| GR3 | 249 | NO | 0.79 | 1 | gustatory receptor | *Tribolium castaneum* | NP_001137600.1 | 2e-13 | 44% |
| GR4 | 1149 | YES | 0.92 | 7 | gustatory receptor Gr109 | *Tribolium castaneum* | NP_001138957.1 | 6e-15 | 21% |
| GR5 | 249 | NO | 1.14 | 1 | gustatory receptor 3 | *Pyrrhalta maculicollis* | APC94248.1 | 2e-29 | 60% |
| GR6 | 1182 | YES | 3.74 | 7 | gustatory receptor 1 | *Monochamus alternatus* | AIX97155.1 | 0.0 | 87% |
| GR7 | 399 | NO | 5.06 | 0 | gustatory receptor 2 | *Tribolium castaneum* | NP_001161916.1 | 8e-62 | 69% |
| GR8 | 246 | NO | 0.81 | 1 | putative gustatory receptor GR9 | *Colaphellus bowringi* | ALR72586.1 | 1e-21 | 51% |
| GR9 | 1269 | YES | 1.97 | 8 | gustatory receptor 20 | *Tribolium castaneum* | EFA05758.1 | 3e-53 | 31% |
| GR10 | 456 | NO | 3.57 | 3 | gustatory receptor candidate 58 | *Tribolium castaneum* | CAL23191.2 | 4e-07 | 36% |
| GR11 | 1185 | YES | 3.8 | 8 | gustatory receptor 160 | *Tribolium castaneum* | EFA12223.1 | 5e-48 | 29% |
| GR12 | 315 | NO | 0.56 | 1 | gustatory receptor 13 | *Pyrrhalta aenescens* | APC94340.1 | 7e-14 | 52% |
| GR13 | 249 | NO | 2.09 | 1 | gustatory receptor 102 | *Tribolium castaneum* | EFA02935.1 | 4e-13 | 45% |
| GR14 | 429 | NO | 0.75 | 2 | gustatory receptor 153 | *Tribolium castaneum* | EFA07631.2 | 1e-09 | 44% |
| GR15 | 900 | NO | 2.08 | 5 | gustatory receptor 68a-like | *Anoplophora glabripennis* | XP_018567270.1 | 9e-20 | 28% |
| GR16 | 579 | NO | 1.4 | 2 | gustatory receptor candidate 55 | *Tribolium castaneum* | CAL23188.2 | 3e-65 | 57% |
| GR17 | 738 | NO | 1.01 | 5 | gustatory receptor | *Tribolium castaneum* | NP_001137599.1 | 1e-14 | 29% |
| IR1 | 978 | NO | 1.04 | 1 | ionotropic receptor IR2 | *Colaphellus bowringi* | ALR72541.1 | 2e-134 | 59% |
| IR2 | 1668 | YES | 2.65 | 4 | ionotropic receptor IR5 | *Colaphellus bowringi* | ALR72540.1 | 0.0 | 56% |
| IR3 | 2772 | YES | 1.37 | 3 | ionotropic receptor IR6 | *Colaphellus bowringi* | ALR72535.1 | 0.0 | 82% |
| IR4 | 339 | NO | 1.23 | 0 | ionotropic receptor 8a | *Colaphellus bowringi* | ALR72538.1 | 5e-25 | 44% |
