# Supplementary material for: Identification and Expression Patterns of Anoplophora chinensis (Forster) Chemosensory Receptor Genes from the Antennal Transcriptome
Source: Front Physiol. 2018 Feb 13;9:90. doi: 10.3389/fphys.2018.00090 (PMC5819563; doi:10.3389/fphys.2018.00090)
Supplement: Table S3 — Comparative overview of Anoplophora chinensis chemosensory receptor genes idenetified in this study and in Wang et al. (2017) study. [file Table3.doc]

**Table S3** Comparative overview of *Anoplophora chinensis* chemosensory receptor genes idenetified in this study and in Wang et al. (2017) study.

| **Genes name in current study** | **Length(aa)** | **Best blasted hits**  **in Wang et al’s study** | **Length(aa)** | **E value** | **Identical** |
| --- | --- | --- | --- | --- | --- |
| AchiGR1 | 100 | AchiGR2 | 358 | 1.00E-26 | 50.00% |
| AchiGR2 | 247 | AchiGR11 | 413 | 0 | 100.00% |
| AchiGR3 | 82 | AchiGR14 | 83 | 5.00E-53 | 90.10% |
| AchiGR4 | 382 | No hits found |  |  |  |
| AchiGR5 | 82 | AchiGR14 | 83 | 2.00E-15 | 40.50% |
| AchiGR6 | 393 | No hits found |  |  |  |
| AchiGR7 | 132 | AchiGR4 | 133 | 7.00E-97 | 100.00% |
| AchiGR8 | 81 | AchiGR14 | 83 | 1.00E-13 | 36.70% |
| AchiGR9 | 422 | AchiGR9 | 146 | 2.00E-106 | 100.00% |
| AchiGR10 | 151 | AchiGR1 | 152 | 2.00E-109 | 100.00% |
| AchiGR11 | 394 | AchiGR6 | 313 | 0 | 98.10% |
| AchiGR12 | 104 | AchiGR14 | 83 | 2.00E-09 | 34.60% |
| AchiGR13 | 82 | AchiGR14 | 83 | 1.00E-53 | 87.80% |
| AchiGR14 | 142 | AchiGR13 | 118 | 8.00E-78 | 94.70% |
| AchiGR15 | 299 | No hits found |  |  |  |
| AchiGR16 | 192 | AchiGR16 | 218 | 2.00E-140 | 100.00% |
| AchiGR17 | 245 | AchiGR1 | 152 | 2.00E-28 | 39.70% |
| AchiIR1 | 326 | AchiIR18 | 303 | 9.00E-161 | 99.10% |
| AchiIR2 | 555 | AchiIR16 | 83 | 1.00E-53 | 100.00% |
| AchiIR3 | 923 | AchiIR11 | 746 | 0 | 99.90% |
| AchiIR4 | 112 | No hits found |  |  |  |
| AchiOR1(Orco) | 477 | AchiOR35 | 465 | 0 | 100.00% |
| AchiOR2 | 127 | AchiOR26 | 198 | 7.00E-94 | 100.00% |
| AchiOR3 | 194 | AchiOR31 | 381 | 4.00E-73 | 54.10% |
| AchiOR4 | 193 | AchiOR14 | 245 | 1.00E-142 | 96.90% |
| AchiOR5 | 95 | AchiOR27 | 395 | 6.00E-69 | 100.00% |
| AchiOR6 | 72 | AchiOR33 | 397 | 8.00E-21 | 52.80% |
| AchiOR7 | 148 | AchiOR28 | 261 | 3.00E-44 | 48.90% |
| AchiOR8 | 221 | AchiOR13 | 131 | 1.00E-46 | 55.60% |
| AchiOR9 | 107 | AchiOR16 | 98 | 1.00E-60 | 96.40% |
| AchiOR10 | 424 | AchiOR32 | 204 | 7.00E-138 | 98.90% |
| AchiOR11 | 309 | AchiOR17 | 389 | 0 | 99.30% |
| AchiOR12 | 402 | AchiOR5 | 79 | 3.00E-52 | 100.00% |
| AchiOR13 | 99 | AchiOR30 | 120 | 2.00E-73 | 100.00% |
| AchiOR14 | 146 | AchiOR32 | 204 | 2.00E-32 | 59.50% |
| AchiOR15 | 141 | AchiOR20 | 158 | 9.00E-21 | 100.00% |
| AchiOR16 | 292 | AchiOR28 | 261 | 3.00E-56 | 40.40% |
| AchiOR17 | 64 | AchiOR23 | 210 | 8.00E-29 | 68.80% |
| AchiOR18 | 49 | No hits found |  |  |  |
| AchiOR19 | 376 | AchiOR31 | 381 | 7.00E-82 | 37.50% |
| AchiOR20 | 114 | AchiOR41 | 386 | 4.00E-81 | 100.00% |
| AchiOR21 | 122 | AchiOR16 | 98 | 2.00E-59 | 79.60% |
| AchiOR22 | 384 | AchiOR34 | 155 | 6.00E-105 | 90.30% |
| AchiOR23 | 283 | AchiOR13 | 131 | 8.00E-94 | 99.20% |
| AchiOR24 | 385 | AchiOR24 | 386 | 0 | 98.40% |
| AchiOR25 | 122 | AchiOR15 | 386 | 3.00E-87 | 99.20% |
| AchiOR26 | 145 | AchiOR13 | 131 | 6.00E-79 | 79.20% |
| AchiOR27 | 55 | No hits found |  |  |  |
| AchiOR28 | 96 | AchiOR19 | 174 | 1.00E-70 | 99.00% |
| AchiOR29 | 312 | AchiOR15 | 386 | 0 | 92.60% |
| AchiOR30 | 366 | AchiOR9 | 423 | 4.00E-29 | 22.80% |
| AchiOR31 | 216 | No hits found |  |  |  |
| AchiOR32 | 384 | AchiOR38 | 385 | 0 | 100.00% |
| AchiOR33 | 357 | AchiOR44 | 275 | 0 | 98.90% |
| AchiOR34 | 145 | AchiOR13 | 131 | 6.00E-79 | 79.20% |
| AchiOR35 | 112 | AchiOR13 | 131 | 4.00E-21 | 34.80% |
| AchiOR36 | 199 | AchiOR31 | 381 | 1.00E-80 | 57.90% |
| AchiOR37 | 152 | AchiOR28 | 261 | 5.00E-90 | 79.50% |
| AchiOR38 | 73 | AchiOR23 | 210 | 2.00E-49 | 91.80% |
| AchiOR39 | 208 | AchiOR9 | 423 | 2.00E-146 | 97.10% |
| AchiOR40 | 107 | AchiOR2 | 108 | 7.00E-78 | 99.10% |
| AchiOR41 | 382 | AchiOR43 | 329 | 0 | 99.40% |
| AchiOR42 | 168 | AchiOR31 | 381 | 1.00E-38 | 41.50% |
| AchiOR43 | 417 | AchiOR3 | 153 | 4.00E-99 | 98.60% |
| AchiOR44 | 435 | AchiOR11 | 436 | 0 | 99.50% |
| AchiOR45 | 156 | No hits found |  |  |  |
| AchiOR46 | 149 | AchiOR6 | 87 | 5.00E-16 | 30.00% |
| AchiOR47 | 379 | AchiOR33 | 397 | 0 | 99.20% |
| AchiOR48 | 389 | AchiOR43 | 329 | 5.00E-68 | 34.70% |
| AchiOR49 | 316 | AchiOR9 | 423 | 0 | 93.40% |
| AchiOR50 | 239 | No hits found |  |  |  |
| AchiOR51 | 380 | AchiOR31 | 381 | 0 | 99.50% |
| AchiOR52 | 375 | AchiOR31 | 381 | 0 | 77.40% |
| AchiOR53 | 104 | AchiOR25 | 161 | 1.00E-75 | 100.00% |

Note: These genes sequences were compared using NCBI protein-protein BLASTP 2.6.0+.
