# Supplementary material for: Identification and Expression Patterns of Anoplophora chinensis (Forster) Chemosensory Receptor Genes from the Antennal Transcriptome
Source: Front Physiol. 2018 Feb 13;9:90. doi: 10.3389/fphys.2018.00090 (PMC5819563; doi:10.3389/fphys.2018.00090)
Supplement: Table S4 — Comparative overview of Anoplophora chinensis chemosensory receptor genes identified in this study and Anoplophora glabripennis chemosensory receptor genes reported in Hu et al. (2016) study. [file Table4.doc]

**Table S4** Comparative overview of *Anoplophora chinensis* chemosensory receptor genes identified in this study and *Anoplophora glabripennis* chemosensory receptor genes reported in Hu et al. (2016) study.

| **Genes name in current study** | **Length(aa)** | **Best Blasted hits**  **in Hu et al’s study** | **Length(aa)** | **E value** | **Identical** |
| --- | --- | --- | --- | --- | --- |
| AchiGR1 | 100 | AglaGR3 | 116 | 1.00E-07 | 42.90% |
| AchiGR2 | 247 | No hits found |  |  |  |
| AchiGR3 | 82 | AglaGR6 | 261 | 1.00E-12 | 37.80% |
| AchiGR4 | 382 | AglaGR11 | 106 | 3.00E-73 | 98.10% |
| AchiGR5 | 82 | AglaGR7 | 131 | 2.00E-17 | 34.60% |
| AchiGR6 | 393 | No hits found |  |  |  |
| AchiGR7 | 132 | No hits found |  |  |  |
| AchiGR8 | 81 | AglaGR7 | 131 | 1.00E-11 | 37.70% |
| AchiGR9 | 422 | No hits found |  |  |  |
| AchiGR10 | 151 | AglaGR6 | 261 | 3.00E-28 | 39.60% |
| AchiGR11 | 394 | No hits found |  |  |  |
| AchiGR12 | 104 | No hits found |  |  |  |
| AchiGR13 | 82 | AglaGR6 | 261 | 1.00E-12 | 37.80% |
| AchiGR14 | 142 | No hits found |  |  |  |
| AchiGR15 | 299 | No hits found |  |  |  |
| AchiGR16 | 192 | No hits found |  |  |  |
| AchiGR17 | 245 | AglaGR6 | 261 | 5.00E-27 | 27.70% |
| AchiIR1 | 326 | No hits found |  |  |  |
| AchiIR2 | 555 | AglaIR1 | 239 | 4.00E-07 | 22.00% |
| AchiIR3 | 923 | AglaIR1 | 239 | 3.00E-09 | 23.60% |
| AchiIR4 | 112 | No hits found |  |  |  |
| AchiOR1(Orco) | 477 | AglaOR31 | 97 | 1.00E-07 | 32.10% |
| AchiOR2 | 127 | AglaOR8 | 331 | 2.00E-20 | 35.80% |
| AchiOR3 | 194 | AglaOR23 | 87 | 6.00E-25 | 41.20% |
| AchiOR4 | 193 | AglaOR34 | 287 | 2.00E-12 | 27.40% |
| AchiOR5 | 95 | AglaOR34 | 287 | 4.00E-15 | 32.20% |
| AchiOR6 | 72 | AglaOR26 | 104 | 5.00E-10 | 37.30% |
| AchiOR7 | 148 | AglaOR27 | 161 | 3.00E-07 | 22.70% |
| AchiOR8 | 221 | AglaOR16 | 199 | 4.00E-149 | 99.00% |
| AchiOR9 | 107 | AglaOR8 | 331 | 1.00E-69 | 90.70% |
| AchiOR10 | 424 | AglaOR8 | 331 | 2.00E-107 | 45.30% |
| AchiOR11 | 309 | AglaOR17 | 152 | 6.00E-08 | 29.30% |
| AchiOR12 | 402 | AglaOR12 | 123 | 7.00E-07 | 31.50% |
| AchiOR13 | 99 | AglaOR15 | 385 | 2.00E-22 | 40.70% |
| AchiOR14 | 146 | AglaOR8 | 331 | 3.00E-106 | 95.90% |
| AchiOR15 | 141 | No hits found |  |  |  |
| AchiOR16 | 292 | AglaOR12 | 123 | 1.00E-17 | 53.60% |
| AchiOR17 | 64 | AglaOR12 | 123 | 3.00E-30 | 69.40% |
| AchiOR18 | 49 | No hits found |  |  |  |
| AchiOR19 | 376 | AglaOR27 | 161 | 1.00E-115 | 96.90% |
| AchiOR20 | 114 | AglaOR15 | 385 | 2.00E-67 | 82.50% |
| AchiOR21 | 122 | AglaOR8 | 331 | 4.00E-67 | 82.30% |
| AchiOR22 | 384 | AglaOR21 | 119 | 3.00E-79 | 89.90% |
| AchiOR23 | 283 | AglaOR16 | 199 | 5.00E-75 | 54.60% |
| AchiOR24 | 385 | AglaOR15 | 385 | 0 | 91.40% |
| AchiOR25 | 122 | AglaOR8 | 331 | 9.00E-18 | 33.60% |
| AchiOR26 | 145 | AglaOR16 | 199 | 2.00E-55 | 53.10% |
| AchiOR27 | 55 | No hits found |  |  |  |
| AchiOR28 | 96 | AglaOR8 | 331 | 7.00E-20 | 39.00% |
| AchiOR29 | 312 | AglaOR15 | 385 | 1.00E-10 | 28.70% |
| AchiOR30 | 366 | AglaOR17 | 152 | 1.00E-19 | 31.60% |
| AchiOR31 | 216 | AglaOR16 | 199 | 3.00E-09 | 57.10% |
| AchiOR32 | 384 | AglaOR15 | 385 | 2.00E-61 | 30.00% |
| AchiOR33 | 357 | AglaOR32 | 139 | 1.00E-90 | 93.50% |
| AchiOR34 | 145 | AglaOR16 | 199 | 2.00E-55 | 53.10% |
| AchiOR35 | 112 | AglaOR17 | 152 | 5.00E-19 | 35.80% |
| AchiOR36 | 199 | AglaOR23 | 87 | 6.00E-28 | 47.10% |
| AchiOR37 | 152 | AglaOR4 | 74 | 8.00E-48 | 89.20% |
| AchiOR38 | 73 | AglaOR12 | 123 | 1.00E-48 | 89.00% |
| AchiOR39 | 208 | AglaOR17 | 152 | 9.00E-101 | 91.50% |
| AchiOR40 | 107 | AglaOR16 | 199 | 3.00E-18 | 33.70% |
| AchiOR41 | 382 | AglaOR16 | 199 | 1.00E-21 | 30.30% |
| AchiOR42 | 168 | AglaOR27 | 161 | 6.00E-12 | 36.60% |
| AchiOR43 | 417 | AglaOR29 | 127 | 9.00E-92 | 100.00% |
| AchiOR44 | 435 | AglaOR16 | 199 | 1.00E-25 | 35.40% |
| AchiOR45 | 156 | AglaOR8 | 331 | 9.00E-06 | 22.70% |
| AchiOR46 | 149 | AglaOR17 | 152 | 3.00E-08 | 28.60% |
| AchiOR47 | 379 | AglaOR17 | 152 | 1.00E-17 | 28.20% |
| AchiOR48 | 389 | AglaOR17 | 152 | 8.00E-28 | 31.00% |
| AchiOR49 | 316 | AglaOR17 | 152 | 5.00E-107 | 97.40% |
| AchiOR50 | 239 | No hits found |  |  |  |
| AchiOR51 | 380 | AglaOR23 | 87 | 6.00E-28 | 50.60% |
| AchiOR52 | 375 | AglaOR23 | 87 | 1.00E-25 | 50.60% |
| AchiOR53 | 104 | AglaOR31 | 97 | 5.00E-33 | 100.00% |

Note: These genes sequences were compared using NCBI protein-protein BLASTP 2.6.0+.
