# Supplementary material for: Identification and Expression Patterns of Anoplophora chinensis (Forster) Chemosensory Receptor Genes from the Antennal Transcriptome
Source: Front Physiol. 2018 Feb 13;9:90. doi: 10.3389/fphys.2018.00090 (PMC5819563; doi:10.3389/fphys.2018.00090)
Supplement: Table S5 — Comparative overview of Anoplophora chinensis odorant receptor genes identified in this study and Anoplophora glabripennis OR genes reported in Mitchell et al. (2017) study. [file Table5.doc]

**Table S5** Comparative overview of *Anoplophora chinensis* odorant receptor genes identified in this study and *Anoplophora glabripennis* OR genes reported in Mitchell et al. (2017) study.

| **Genes name in current study** | **Length(aa)** | **Best blasted hits**  **in Robert et al’s study** | **Length(aa)** | **E value** | **Identical** |
| --- | --- | --- | --- | --- | --- |
| AchiOR1(Orco) | 477 | AglaOR1/Orco | 477 | 0 | 99.60% |
| AchiOR2 | 127 | AglaOR50 | 391 | 4.00E-89 | 96.90% |
| AchiOR3 | 194 | AglaOR74 | 376 | 1.00E-126 | 91.20% |
| AchiOR4 | 193 | AglaOR18NTE | 388 | 1.00E-141 | 96.90% |
| AchiOR5 | 95 | AglaOR20NTE | 162 | 7.00E-53 | 79.80% |
| AchiOR6 | 72 | RoAglaOR14 | 396 | 8.00E-36 | 73.60% |
| AchiOR7 | 148 | RoAglaOR85 | 359 | 2.00E-101 | 92.60% |
| AchiOR8 | 221 | RoAglaOR33 | 418 | 7.00E-164 | 99.10% |
| AchiOR9 | 107 | RoAglaOR46NTE | 401 | 8.00E-68 | 90.70% |
| AchiOR10 | 424 | RoAglaOR43CTE | 388 | 0 | 86.60% |
| AchiOR11 | 309 | RoAglaOR19 | 386 | 0 | 95.30% |
| AchiOR12 | 402 | RoAglaOR102 | 402 | 0 | 92.50% |
| AchiOR13 | 99 | RoAglaOR55 | 393 | 2.00E-68 | 98.00% |
| AchiOR14 | 146 | RoAglaOR46NTE | 401 | 2.00E-104 | 95.90% |
| AchiOR15 | 141 | RoAglaOR101 | 404 | 1.00E-102 | 97.90% |
| AchiOR16 | 292 | RoAglaOR100 | 359 | 0 | 96.90% |
| AchiOR17 | 64 | RoAglaOR100 | 359 | 9.00E-37 | 87.50% |
| AchiOR18 | 49 | No hits found |  |  |  |
| AchiOR19 | 376 | RoAglaOR79 | 376 | 0 | 96.80% |
| AchiOR20 | 114 | RoAglaOR60 | 385 | 6.00E-81 | 100.00% |
| AchiOR21 | 122 | RoAglaOR44 | 403 | 6.00E-77 | 94.70% |
| AchiOR22 | 384 | RoAglaOR68 | 384 | 0 | 98.40% |
| AchiOR23 | 283 | RoAglaOR36 | 421 | 0 | 96.80% |
| AchiOR24 | 385 | RoAglaOR61NTE | 321 | 0 | 91.60% |
| AchiOR25 | 122 | RoAglaOR51 | 385 | 4.00E-84 | 95.90% |
| AchiOR26 | 145 | RoAglaOR35 | 421 | 9.00E-101 | 97.20% |
| AchiOR27 | 55 | No hits found |  |  |  |
| AchiOR28 | 96 | RoAglaOR48 | 405 | 6.00E-61 | 96.60% |
| AchiOR29 | 312 | RoAglaOR51 | 385 | 0 | 90.10% |
| AchiOR30 | 366 | RoAglaOR8 | 402 | 0 | 98.10% |
| AchiOR31 | 216 | RoAglaOR35 | 421 | 4.00E-156 | 97.20% |
| AchiOR32 | 384 | RoAglaOR63CTE | 368 | 0 | 96.50% |
| AchiOR33 | 357 | RoAglaOR72 | 406 | 0 | 94.70% |
| AchiOR34 | 145 | RoAglaOR35 | 421 | 9.00E-101 | 97.20% |
| AchiOR35 | 112 | RoAglaOR11 | 384 | 1.00E-70 | 89.30% |
| AchiOR36 | 199 | RoAglaOR73 | 381 | 3.00E-128 | 92.00% |
| AchiOR37 | 152 | RoAglaOR97 | 359 | 2.00E-97 | 88.80% |
| AchiOR38 | 73 | RoAglaOR86 | 359 | 1.00E-45 | 89.00% |
| AchiOR39 | 208 | RoAglaOR3 | 400 | 3.00E-137 | 92.30% |
| AchiOR40 | 107 | RoAglaOR16 | 379 | 6.00E-74 | 96.30% |
| AchiOR41 | 382 | RoAglaOR28 | 381 | 0 | 86.40% |
| AchiOR42 | 168 | RoAglaOR74 | 376 | 2.00E-113 | 90.50% |
| AchiOR43 | 417 | RoAglaOR30 | 417 | 0 | 97.80% |
| AchiOR44 | 435 | RoAglaOR29 | 435 | 0 | 97.50% |
| AchiOR45 | 156 | RoAglaOR37 | 402 | 4.00E-91 | 89.70% |
| AchiOR46 | 149 | RoAglaOR37 | 402 | 3.00E-98 | 92.60% |
| AchiOR47 | 379 | RoAglaOR15CTE | 381 | 0 | 96.70% |
| AchiOR48 | 389 | RoAglaOR27CTE | 367 | 0 | 95.10% |
| AchiOR49 | 316 | RoAglaOR3 | 400 | 0 | 96.80% |
| AchiOR50 | 239 | RoAglaOR16 | 379 | 2.00E-166 | 93.30% |
| AchiOR51 | 380 | RoAglaOR78 | 380 | 0 | 93.90% |
| AchiOR52 | 375 | RoAglaOR77 | 375 | 0 | 97.10% |
| AchiOR53 | 104 | RoAglaOR6 | 403 | 2.00E-72 | 100.00% |

Note: These genes sequences were compared using NCBI protein-protein BLASTP 2.6.0+.
