# Supplementary material for: Identification and Expression Patterns of Anoplophora chinensis (Forster) Chemosensory Receptor Genes from the Antennal Transcriptome
Source: Front Physiol. 2018 Feb 13;9:90. doi: 10.3389/fphys.2018.00090 (PMC5819563; doi:10.3389/fphys.2018.00090)
Supplement: File S1 — The amino acid sequences of Anoplophora chinensis putative chemosensory receptor genes. [file DataSheet1.DOCX]

>AchiOR1 (Orco)

MMKFKVSGLVADLMPNIRLIQASGHFMFNYHADNSGALHALRLGYSCAHLLFCLFQYGCIFGNLVVEKDDVNYLAANTITVLFFTHCITKFVYFALRSKLFYRTLGIWNQSNSHPLFVESNNRYHALALKKMRTLLICVTATTVLSAAAWTGITFVEESVHNIKDPDNENETITEEIPRLLVKSWYPWDAMSGMAYYGSLIFQIYYVLFSLAHANLMDSLFCSWLIFACEQLQHLKEIMKPLMELSASLDTYVPKSADLFRAPSAKSQDNYIENDYNAKNEELNLKGIYNTRQELGGNFRTGALQTFGQGGVGPNGLTKKQELMVRSAIKYWVERHKHVVRLVTAIGDAYGVALLLHMLTSTVMLTLLAYQATKINGVNTYAATTIGYLVYSLAQVFHFCIFGNRLIEESSSVMEAAYSCHWYDGSEEAKTFVQIVCQQCQKAMQISGAKFFTISLDLFASVLGAVVTYFMVLVQLK

>AchiOR2

MLLEFLITSGELSMFIFIVIMTSSPNSKMFSGACIVNLLVKILILCWHADQIREESIGIADAVYELPWLEYDKSDVTSLQIIMMRSQKPLTLTIGPFGTMTLELAVKIFKATYTYVTFMQNVYGKLD

>AchiOR3

MFVLTLLLLTKMQFKLLNQEMERLFNEMSSSEGKSKHFDKKIKKVVEYHDFLLDFVDRINNGFSEVLLVYIIVLVLGMCVQMYNVSTQPTLMGLIRPIVRLINGLTQFIFCYCLPAQAVTDEADRTTEYAYFNRWYDNPSSAKAAQLMILGRSQRKTHILAGGIVNIDLDNCLKTIKTMVSYAMFIRTMGIGQD

>AchiOR4

MMDLCATQFEILKERLIRIGTSFTGDETIDDKLRLRKLRKYIIHHNYIYSCSELVRDTYSIGEFCQVGCSVMVVCFGLFKLLIIPLKSAQFLMLVTYSTTMIYQVSLYCCYGQKLLNASNTITEACYMSRWDDCSTQVQKYLAMIMNRANTPFLMKAGGIFSLTLETLMTIYTSAYSFFAILWKVYHSEDQAP

>AchiOR5

MYSCTMGLEIIFYCCYGQKVLDSSSTISEACFMSNWHHCSIKVQKNMVLLMSRANRSVTMQAGGMFPLTLETLMSIWRSAYSFLTFLMQVYKEDN

>AchiOR6

MVFVQMLGTIVVMCVCCLRFSADEPLSTAFFGTITFTIAALMEMFLYCYSGTLLYEESNSIINDIYMSEWYT

>AchiOR7

MRHFWNTDVEDDQVTYYVQYFKTIEKVYKHAIFFGIILHVAKPFFVRGSSICNCYIPPQIPFPIFYAFEFYAIIVGAASAFCFNVFVCSLIVSVAAQFRLVNLKIKDLNAMEIDNDHDLRVYKVNLKSIIKYQQFLIRFVDDINKLLS

>AchiOR8

MNVSLDVFAWIIGTHDGFFVTLMNCLRTQLEIVQCAFRTIRERCEKKLDLPENYKIFVDESNPTLEKTLYDELTHCTKHLNILLQVRDDIENVFTYVTLAQTLASLIIFASCLYVASTVSMTSPEFFAQVEYFLCVLVQLSVICFFGNEITTASAQTGVSLYECDWFSSSLRFKRSMILTMSRIQRPVYVSIGKFSPLTLATLVAVCRGSFSYFTLFKSVQ

>AchiOR9

MIQFYLAKTTVYNTIFHGSFAFSMLLRLLVYYWYANEIMLESFNVSTAIYECGWYDEPQKVKQMMLLMIQRANKVLKLDIGPFTTMTLGTFLSILKASYSYLTVMYR

>AchiOR10

MIIPKNEYLKLAMYISAVLGVWPFIFEDKPVLRKIYDVYSKCLFCYYIFYVLTAIIQLFIVVTDEVLDVSEIVSNLCITLLNFIAILRVKAIKSERVKNIIQNVFKLEEKMMNSGNDEIIEMYNRYARQNQICNKIFVVNIYLVDILYYIHPLYVEDTIKYYPKRNETVVIKALPLSTWFPFDQQKHYVLSYIWQVTDIFMATTFISCADIFAFSLILFGVGQIKILKLILSNFQEYTMKIKNLLHCSQEEASYITLRECILKHQEIIEYIKEYNLVMKNIMVLDFLMSSMQLASIVLTLLVTKVTLINTIYSGQFAVCMFLRLLVYYWYANEIMVHGSEIGLALCNSNWYEESERVQKMMVIMLMRCNRELCLEIGPFAAMTLRTFLGVSRREQYKIGNHFYNINEIWVIKSVNGRYLSMIGF

>AchiOR11

MVFGGFYSTKKYKTLHNLSAILIMAVVSLYNLLGLLHGFQHISNVAVFSQSIAYLLTGISFSCKMINLIVHKNNLLLLDEILQNPVSTELETTEEEVVLRNKLKFGQTLKKIYKLYTSATVTVQVLYPMINNPGHKNFPLLFWFPFNPEDHYYKVYFAEILMIFCICTFNVTVDLLNVLFMDLCAAQFELLKYRLKHFGREFHGGEAVNDRTLYEKLNKIIVHQNLVYRFSKLTEDTFSAGVFCHLACTVVVLCCAIFKAVITPIKSMQFLMMATYSFCMTLEVSLYCCYGQKVLDSVRQLCIVKKNRI

>AchiOR12

MVIKFPKPGEFILRDDHMASMRKFLKMHDFDGKTKYFWISVISLEILLLVSRITAIFQCLDRPRRVVDLSATIPITITALLKAIYLYYDKNRVKYLYNTIESEFWDFRICGPEVQKQAITRYKYSNRVLWNLLTNGIICYLLLQIFPMTPMPSESDRMLPKVVWSPVDLNPSPLHEIAFAIILLNGLLTALSNMIYDYFYVYCAQHLVVQLIILQNLLRNITEDVMVHLSDVEKFNSENFQDTVMNRMKICAEQHSKLLKYGENLGRFCSLVLVPQLALTYAGLVVSGFIIATDGSSFYQDVIYFSLSLNTLLELSTFAIPSSHMNTQSLLVLDAIYDSKWYLFNSRMKRACTFMMMNGQEGISVKAGGITKIDNSLLVDMMQKVFSAITLMTALIDANQTN

>AchiOR13

MDKLRYFLHLMGWIGMLFITCFYGQMILDESVTIADAAYQSEWYNGPEHLKKMICLIIVRSQRPLLLRVASIGVVSLETFVSVIKTAYSYFALLLTIAK

>AchiOR14

MAAILIFIHSLHEENKIKFDKATNETKIIKTLPLSSWLPYDPQDHYLISYLWLTFDGMVGAFYMMYTDAYNFNLIIFPLGQIRILTHVLSNFPRYVLKVKDQLQCSRDEASFVTLRECILKHKEIIRYLQEYNDSVKNIMLLDFLQ

>AchiOR15

MEMPKGVKRLPNPVWTPFDTNVSPLYEILYFLLVCSQVLTVFGNGYYDFAYGSATQHLCAQLLLLKEQLKNITVGIMPHASDLEKFNSGYFQKRVMERLKICVRHHCRLLKYGKNLDRNSSSILLLQLLMSYLAMVINGYI

>AchiOR16

MKNKFCHKNFLFLGAHPFHLLRKWLCIFNIALLIYFLFMALYQFHRDKDLEALETLSCIILVMAKYLTLIFRPKLIRQIVEATENFWELNIIDERVIVFLKHLKRVENIYKYTFVLVLLIFAAKPLVVKGSSVFNCYVPPEIWYPSYYVAEMYTLTVATSAFLSWNVFVCFLIILVVVQFRLLNLNIKALNCEDVQNEHDFDRYVRDLKSVIQYQQNLMRYIKVLNELLHVPVAVLMITSVFLMCLNMYALTTSKGSMVDNGRIMISCSTLIVEFFMVYGLPAQLLMDESEA

>AchiOR17

MIYFAYKWYLSNLRPLRKHFLIMMTRSQKGVYIRAGNYYIINNRTILIMMRTAYSFYTFLQKVA

>AchiOR18

MTRSFHKLLLEGGHISAHGFYLTEIKIERAALCCHFAPISTDKFDEYTK

>AchiOR19

MSFYNINFCLKLFNLMGTHPDKRGSVLQVMIYILGAVLNLSIVFLSILLLFIKERVTITDVTDSIETIGLLLHGFLKLTNLFIKRSKISNLLRAMEAHFWKIEDVTDTDARTSHQRFLNSLKNVLKFFTSLCAFLAVLFIIKGFVGQTVFEIYLPNWFPLYLSVFYQSFTCVLTISFPVISTDLFIFTIFMLTSLQFKLLNEEARNVFGKKGEGGIDDEVIKARIKKCVDHHIFLKDFVKMLNDTFSQVLFIYNGDIVLSLCVEMFIVSTQNSFQTAAKAAVYVFTGMFQYTVCYCIAAQAITDEASEISKSVYFGNWYEHPEKYIRNATTLMIGVGQRPVAITALSFIKVNLETCLKTTQTILSYCMFLRTMGIN

>AchiOR20

MACALVFGCIGNLILKSKPIAGISYLLEYVFALFLLCHGGQRLLDETLSVADVIYDTKWYDGDVQIMRDIRFILARSQIPVMIGALPLGCMNYALFLMIMKTSYSYLTLLTQNT

>AchiOR21

MKNIMLLDFLQSSLQLASIVIQLFVTKATVFNIIFHGEFTICMLIRLLVYYWYANEIMLESINVSTAVYECGWYEEPQKVKQMMLLVIQRANKALELEIGPFTTMTLRSFLGVSLFYIYIKY

>AchiOR22

MAKPQIVCLQNTLKIFRFYAMFPEKDKELNHGKNHYVKFAVLACFSSVVLIGSFLHLIKSMSDNNYLNLDTDLMYTLSLGITFAFSIGFLYKTEALVEIFLFLSDFEEFGKPVDFDRDNTFYDRLSVYHYIYLETLVSGIVIISTYLNMTKCQKLNLEYGLDEICGLFSYTWMPFEIDYFPMRQIFLVVQMFGAHQFYMIAGMCAWFVFETFQHFRIRIRHVNFLFQEALKEEDPQRCREKFNHAARYHALLLGLGDRMNGAFGIFMFTHMAITAPIIGIGVFAIVSGGSVSSFLLCLGWFDGLAMVCFAGQWLQDECFAVGTDLYDADWLHCPEDIKKDMMVVIQRSWTPICLRASSFGIMDYRMFLGVLKASYSYITLLTQT

>AchiOR23

MYCIYAFVGLSAHVSVAATIRNQVNGTKFEGEYSCQDFVPYYFLIPFEVDTTTKCDYVLFFMDISALFYGTLIGCYDGFLAALLNCVKTQLLILQDAIRSIRERVMQRLALDERLDIFHDETLPKLEEELYKEFKHLVKHLTIILRVADELESMYNLMILAQTLTSLFNVASCLFSASREPVGSAVFFATLVYFTSILIELGVVCWFGGEITTASEDIMFALYEVDWFSASQRFKHSLILTMCRMQRPIYLSIGKFFPLTLSAMVSVCKASFSYYTVFRRSDE

>AchiOR24

MYEITKERSFYSSLKILTHTYVIPKVKEGPQLFRFRFIFIMLRLASITTFVVMPCLHLIVTLRAKTGVDVSEDFSTMFGTFGCNVVTISFLLLYDKWSQFFIDLENCNRFGIPADIGALRKKLNLYSVLYALYTVFGVVTYGMIATAETSHCKKFNEEHGLNEICGTFTPLVLPFDGNPIFVRSLIFAIQVFLAVTTLSAPAVVCFMLYEGVETLIAYITLLKKNFINVFETSSEAERKDKLRFCVYFHDNMLSLCQRLSTLGKYSAGFLCMTCAVVFGCIGNLILKSKPVAGICYFLGYIAALFLLCHGGQRILDETLSVADVLYDSKWYDGDVQIMRDICFILARAQIPATLGALPLGVMNYPLFLMIMKTSYSYLTLLSQNT

>AchiOR25

MLMEFLFASLRLALSIFLLVTANTPNSKIYFGTVIVNVLINILILCWNADQIREESIGIADSIYQLPWFEYDKSDVISLHIMMIRSQTPLTLTTGPFGTVTLDLAGKIVKATYTYATFMHQM

>AchiOR26

MHLNIILSVTEEIEKMFNLMILAQTLSSLFNIASCLFTAAKEPVGSIMFFATLTYFLSMLIEFALVCWFGSEITTASEELMFALYDIDWFSASHRFKKSLLLTMNRMQRPIYLSIGKFFPLTLKATVSVCKASFSYYTVFRRVDD

>AchiOR27

MKPWLWNIKLENIQNNNFARARNKSELIYFIDTCPVEGCFLSRELLCFINSLILY

>AchiOR28

MLLVHMLLFYWHADEIRHESMAISEALYETDWYEYSRSTSSTIHLMMMRSQRPLSLSVGPFGEMSLTMALKILKGVYTYMTFLQHSYGQTSSLGTN

>AchiOR29

MILYVNLAITTSGNFQKLIYEMIAYEAEVVDVGTEKHLKIHEQVVKKSRTLQVYYISILASCSIAFVVPAFVEYALVQSSEESTNSTINTHNYDMWIPFEESKEYLVWLVVQSCYTFIVTCIYCNYQIILINLLLVVILRLKILRASIENMKDTDALDVKRLIRIYAKEHMDLIRNCKYVDDTVKYVMLLEFLIASFRLALSIFLLVTTNTLNSKICFGTVIVNVLLNILILCWNADQIRQESIGIADSIYQLPWFEYDKSDVMSLHIMMIRSQTPLTLTTGPFGTVTLDLAGKIVKATYTYATFMHQMYEN

>AchiOR30

MKETLNFKEIITLNVEVLNGFGYIAPEFQHKIEAVWFGIRSAVFIGFIYLGMLTSEIVNMILVSGDMEKMIEATFLTLTHLVEIRKVYAVIRYRDRLNKLLNSINRKEFLPKTTTQAKALQDYVQDSKVISKVFLGACVATCSFWGIYPFVDDGDLRLPLGGWFPFDTRYSPWFELAYVYQVIGSTVNGLVNVSLDTFMSGLIMVVCAQLNILNDSLKNMREQAEIELKGVGIVVGQYMTNTLQEKMNEKLLECVNHHRYIIEFANELTFLFTTSILGQFIVSVVIICITLFEITLLPALSIKFFSLILYQFCMLLEIFLLCYYGNEVIRESAELTKFAFCSDWMDCSPEFKRNLVFFMTRSQMAL

>AchiOR31

MIWILIEYTVGTIFLILQFLILSETSKDINKFFSHIGLLFSNALGTVKIAFIILRRNKFKKLMDILHDENYQYESLDEFKPSLIFLKEKRFSKVISISVLCTFTLVGISAHASAVRIIRKEINGTRFEGDYNCQDFVPYYFLIPFEVDTTTKCEYVLFFMDISVCAFAWLVGCYDGFIAALLNCVKTHLVILKGAIRTIRKRVMRRLDLDENLQIF

>AchiOR32

MEADDIVIMENSLRYLSRNLIFPKKKDVNNPGVIFHLKFIALNLSTVMFLTGNILHLTINIKRKTYINLDLDIALAISLFGSYYFNFSYIRQVKNTINIYKQLSDLRSYGIPKDFYATNKKLNNYSKYHYIYIVSAVLGLSVAPLLEYKKCQKENVVKNINEICGLIGSIWLPIDLDSIPYKQMYYIFQVYSSFVIYQTSSLISFSIMETAEHLILRLNHVKHGFLDALMEREDRVRREKFSRAVKYHVNIIRISKLLNTSVSTIMFGHVLLTGAILGCIEYRLLKSYSLGAICLFVGWLISMSMVCIGGQRLRDQSLSIGDAICKSNWFDVNKELQRDLVLVILRCQKPIFIDAGPFGYMTYAMILTVLKTSYSYLTLLSSTS

>AchiOR33

MFTEYGLYSFISNNFKNFNESFTAITSFSTLMQVIVKESIFYYYLKTAKRILDVIFYKFWPSNLVGREVESSLNRYYHILLIGMMSIYFHGSIFSITCILIPLFKETRETPYKTVYPFEYSSSPKFEIMYLMQSFINFYVILGVIIGVDCLFMATCYNIIAQFRLLKGVVLKLGTNEVKEINSKLTILSSESDNIGRNVTEEKEFLIRCIKHHQLLLRTTEDIETVYSVIGLFQLGFSIIAICMSSFVVTTGEIEYVQLVNISIFISGHIVQLFCYCSVANEISFEMDNLSQHIFSSYWYQTDFVNIKQDILVIMKKSQEVKRITALKLVPLNYDTFIQVLRISFSFHTLLSNITVK

>AchiOR34

MHLNIILSVTEEIEKMFNLMILAQTLSSLFNIASCLFTAAKEPVGSIMFFATLTYFLSMLIEFALVCWFGSEITTASEELMFALYDIDWFSASHRFKKSLLLTMNRMQRPIYLSIGKFFPLTLKATVSVCKASFSYYTVFRRVDD

>AchiOR35

MTNMSMNHDVNADFWVLSVLQLAVWVDLYIYCWYGNELTEKSKKIPYAAFESNWVTASKGYKKNLLIFICRTQTPIKLYAVNFFELSLNTFISILRKAYSYYMLLGQLSADE

>AchiOR36

MDKFMLTLLLLTKLQFKLLNQEIEKVFNEMSSSEGKSKHFDKRIKKIVEYHDFLLDFVNRINDVFSEGLLVYVIIIVLSMCIQMYIVSTEHSLMALMKPLFYLTNAMIQYIFCYCLPAQAVTDEADRTTEYAYFSRWYENPSSAKVAQLMILGRSQRKTYILAGGIVKIDLETCLKTIRTMVSYAMFIRTMGIGQDQTR

>AchiOR37

MKKIIKHQQLLMRYVDDLNTILSVPVALLMVTNISIMCITMYNATVTEGNLLVQVRLIMIIVCILTESFFVYGFPAQVMMDQSEATAETIYSECIWYLPKLLHLRNDFLIMMIRSQERVYIRAVNYHIINNRTVLIIMKTAYTFYTFMQKVA

>AchiOR38

MDKSTATADIIYSECKWYIPKLRCLRSYFLIMMTRSQRGVCIRAGNYHVINNRTVLLMAKTAYSFYAFLQNVT

>AchiOR39

MIILALLIQVGLQCDLLCCTLDSLDDFYTEGNVLYEISLEDKLKLRKDRERFSKEMTKNLVICVEHHRQIIRVVKDVERISGTGLFILFVGGGLILCSSLFPLSVVKIGSIEFIMLLFYLICMLTVQFLYCWLGNEIIFKSSLILQSAFNTPWIGCNVKFQKILLVFMMKTSKPMSILTGGLFTMSVPVFVSILRTTYSYFTLLKNIQ

>AchiOR40

MTPLFSGQFVILIVYFSAIMIELAMYCWVGNEIILKSLQIGDACYMSKWYEFSPRTNKILFLIMERSRRPLTISAYKFSVLSMSAYLKIIQCSYSYFTVLRRVYMKD

>AchiOR41

MSTIEDYDLRNAFNIERKLLLLCGIYPNEGRINKKLYNLSAFYHISFSLLITSSMVIFVAMNMNMNLLSIVEALLFLATQMAFLCKLFNVLNKKHKLLEIEDILANPAFYGYPKEKRHFIEDSVRFTKILGICYRSICAMGFIIYGCFQLMKDEEWPLPLSGWNPIQVNTKFKYGIIATFQWVAFFMSAYINSGIDILIYMLISVVTSQFEILKDNLTNIRYETDTAKRDFAKNVVLHYGILKFVRVIEDTFSYATFFQFFTSVIVICFAGFEIMIAPLNSIQFVSMCTYFSIMIFQVVIYCWFGHTIIASSDKINDAIYMSNWYEADLSLKKSIMIFMEKCKEPVVLTAGKICPLSLGTLTNIMRSSYSYLAVLQSIYGQE

>AchiOR42

MYFSLFNMCIILFNRMGVNPMKRYTMPQVLMYITTLSLQMSIIYLGMLLLVYKENVAITDVTNVMDSFMLISHGITKCTLVFVKRNNIRDLLDRIGNFWKIEDVQDEVERKEHQKYLKFIKTMSFLYNFLCICTTITFSCKPLFGELSFNTYRPERIPFHLLQVYESI

>AchiOR43

MEEQYAEDFFVANRWILQCAGLWSPDGQHKVLRMLYKLYSIGIFLFVNIFFTATEFVSLYYTYGNEYDLIKNISFALTHLMGAIKVVFFYFQKHKLLAIMDTLENKEFRYDSCTEKSFFPGLISRRYKNIGVKYTILFFILAHATLFSSYLPPTISTLMNSSRRGAEGQGLYVLPYYSWMPFKYNTDDSFLLALGYQAIPMFSYAYSIVGMDTLFMNILNSIGFNLKMVQGAFLTIRNRIVKQTGKSIYKSSILLDSEELKLKLCNEMKKICHHLQIIYKVCEDLENVHKYLTLAQMTATLFILCSCLYLVSSTPITSKQFYAEIVYMVAMGFQLTLYCWFGNEVTLKAAELPLYIWQCDWLTANNNFKTSMILTMARAKKPLYLSAGNFAPLTLSTFVSIIKGSYSFFTVLKSTNE

>AchiOR44

MELKTPYFKKHLKWLLVLGVDIIPVKNVWYKYFYKLWSVLIIGFVVLYTLLEVIDILNTSDFNSMTFGLCYSATHLLGLAKIIILIVKKKKVREMLNELESGDFLPNMERGGEEEIRLINIAVTRCARHAEIFNLIVYSIVSIRCLYALFDTGYNDELFDEQLNTTTPIHTRILPYRIWLPIETTKSPIFEIVFFFQAFTLTLYGYYIGMMDSMVYGMMFHMNTQYLILKRVLERYVSIATHMVSKNLLDKGVKDIRNGVISLPVGYERIDLLSEPVQEKVREIVHNCAKHHVHILEFCEKVEKEFSYLMLSQFLFSLYTLCFQLYQLSLMANVFSFDFISMCCYLTLLMYQLFCYCFYGNEIMVQSEKFSEALYNSDWLVLHNSTKRSLLLMMMRAQRPIRFTAGKFALLSLQTFMAIVRGSASYFMVLRQMSG

>AchiOR45

MKIISTKERLIINSNDSGIKNVYRSCLQHNTTVFYIALSVIVAVAVLFAVTIYFEVKSWPAAPEGISNCSVSGRELPLPQAVPFGIDKYFELVYVFQVSSRLFAAFIFLGCDTLINYLIYFPAAYIKVLGHKFEHIGDQNGSGLYSPEAFLKKLIL

>AchiOR46

MIEYVIHLNDILQWILSMDFFILSYHISLTLMGIISLLESPAFNMDFMITLCFSVSYLMVVFFQMCLLYYNCNELIVESLEIPNSIFRSNWHELPHAVQRSLLIVIMRTQKPLEMKIGNIYTMSNDLIVGFVKAGFTYVLLSHLDFQKA

>AchiOR47

MKIFGLYPFDSWPQYFTPYALFLYIIFTIATPILAVIHLIVDEKPIVDVITENAFMIVELIALIAKFLPFKIYPERTKKAFSALNKEIFNNHLPEQESVLDETVANCRFIFRIFCTSCAFAVLSWASLPLMYEERRFPIDVWLPFEPFENTAVYLSLYLFVCLSGVHAGFDNATVDSIVALLIYNASSQVIILKDTLMHLSKRTEDEISKENRSLSTEEKENLKSDIIYKKICHCVDHYNAIYQFVEDLEDIFSMVVFSQLIASIIIICICCLQLSVAVPFTIPFFGAVSFLTAALLELFLYCYSGTLLFEESGTIVTAIYMSNWYNYDRKSKKALLTMMERAKRPMMVTAGKLMNFSLETFSTVIRRSYSLLAVLKNY

>AchiOR48

MMETFNMLDFFAAEKLYLTLAGFYPAEPGLRRYLFILSALVNLSISWVQFLSLATFSYFNLNDLRKLTDILVFCVTQFAFLNKLTNLIWHQSSLKELEALLQKTVFTSVRREEKRILTNHLQGGQLLAKMYRVMCFLGVVLYALFPFLDERSDNEPKFPLPCWFPFNEGDYYYPVFFFEIWSIAISAVLNSSIDLLTIMTMILATAEFKILNRKLTNVASSSGVGGDYDDGVEVRSRLGECIIHYDEALNLVRHIELTFSKGIFVQFFCSVMAICLTGFQMLVISFSSMRFALLVVYLLVMMCQVAMYCWYGHNVMDSSDEVTSACYMSRWNKCSVEVRKSLMIVMERAKKPATIRAGNFFTLNIPTLMTILRSSYSYFALLQRVYGNN

>AchiOR49

MMFTYKNMNCLKVLIKEMNRPLFQVKCQKHYHIAKNTERMYKLMFKSCFYLAALTDVFVMVVPLMGKEKMSSVKGWFPYDYTKPLYFILSYIFQKLVFIWNTFISFNIGMIILALLIQVGLQCDLLCCTLDSLDDFYTEGNVLYEISLEDKLKLTTDRERFSKEMTKNLVICIEHHRQILRVVKDVERISGTGLFILFVGGGLILCSSLFPLSVVKIGSIEFIMLLFYLICMLVEQFCYCWFGNEIIFKSSLILQSAFNTPWIGCNVKFQKILLMFMMNTKPMSILTGGLFTMSVPVFVSILRTAYSYFTLLQNIQ

>AchiOR50

MVLYEIVNAIEVPKNMLIVSGVWPHDSPTFWYRLRKLMSYGGTMLLLALMFLEAVVSISNILNLSYVILLSFEYFSYAVRLMVFHLEKVNVRRLSEWLRDPLLNSYGEEHNEFVKSAIARTNRIGTVFQVSCLLATSFLALTPFLADAIFPIPFSFFQEGFMWYVVYLVQVNGLAVGIWNNAGLDTLNIGYMGVALAELDILIYNIEHSAEACASDVTKSKSDVDEYLYTCYKHHNKIM

>AchiOR51

MYLSELYIPLTMCRCMGVYPEEKYRTGQIFIFLIIFISQWIIIYLAILHLIYKDHITISDITNALETLFLIFHAMIKLSMFFVKESEFHDLLERINYFWKVNDLEDEVERKKHQKYLKLIKIRSSMYNFWASATSVAFMLKPFLLKGDNSIFTTVSPAWIPSGVMTFYEEILFAFGVYGPIAGMDLFVLALLLLTRMQFNMLNQEIQCVFQNMTNSEENVEETNEKIKKIVDHHNFLLDYVNRINNAISEGMFLYIVTILLSMCVEMYIASVQKSVMIAIQAMMYASNGLLQYCICYCLPAQSVTDEAELTANYVYFNNWNEHPLPSIKVAQIMIIARAQQKTLILAGGFIKLDLETYLKTLKTMISYAMFIRTMGIGQD

>AchiOR52

MYLSLLYIPLTMCRFMGVYPEEKYRTGQIFIFLIIFFWQWSIIYLAILHLFYKDHITISDLTTVLETLFLIFQAMMKLSMFFVKERQFYDLLQRINYFWKVDDVQDEVEKKNHLNYLKLIKTRSAVYNIWASATTVAFLLKPLFVKGDNLIFTTATPSWIPFGVMPFYEEVLFVFGVHGPIVGMDLFTLALFLLVKLQFDMLNQEIQRMFQNMKESEINQRIKKIVDHHNFMLDYVNRINNTLSEGMLLYVVNVLLAMCVEMYIASVQKSIMAAIKAIMYASTGVLQYCICYCLPAQAVTDEAELTSNYVYFNNWDKQLVSSVKVAQTMMLARAQQQTLILAGGFIKFDLETYLKTLKTMVSYAMFIRTMGIGQD

>AchiOR53

MEKFSKAMVKNLVVCVQHHREIIKLAKDIQRIKEIGIFVLFASGALVLCTCLFQLSMVQFGSVESMMLLFFSICMLTEQFLYCWFGSDVIYKGSLILQAAYNTP

>AchiGR1

MVIRRVYFIYSFGFLIIRTICVCLFAAEIDCESRNPLDVLTSLPSKIYNIEIERFIVHIAKDPAHLSGGGFFIISRSILLRIAGAIVTYELVLIQMYTIK

>AchiGR2

MVYVIPQENVFNVNIKPKYVKESRENIVNSLKFFVICSQFLGILPVENITVPNKLRFRWKSWKVLHTLFFIGMTTVASTLCLLDWFYVGYVFNSLGITIFYSSALVTLILYVNLARSWPKLMTLWCRIDKIMNNSYGYPKSLNRRFRIVSATYSILALGNFLINTVYKSISIKESTRNEYNINKYYFKSFPQMFRFIPFSAVSATFCCIVHIHTLLTWLINDLFIILISIALALRFKQISERLVRNQ

>AchiGR3

MFSCDLAVKEAEKLVATCYKYQAYFPTFSEEKQELLNFAGQIINNRPVFTAAGFFEVNCRTLFALLGTTTTYFIVILQFNQT

>AchiGR4

MGIKSALSSVWNKMDYLDNFKLFDNVYGILDTSYMIYIAFGMTPYLRIKSPEGNFIYYNMSWQKYGISAWYSFLLLLAIAMTVIDELESHFNSIVNIYLIAQMIFSILTLNIFPARANILLKAYGNCHEAEKTLKRLGFKLDYTSIHKITLYGSLLMIFFASMNLTLYILWEKTIFDFIKFCFTYSFPCAVSVIPVYHFNYLGILVLQRLRLVNSYLAEDFSSLTRKQVEYNLWAIAKVHSELCNAVNNVLYASAFSMVTFYFFTYLQFSALLLGAMNIAHLTFDVETYIWNTIFIATTVGILVLATVLKYEATKTSYTLCEQTSTFFDDCIGTRRNTLLMQLLHQKVDLRIFGHFEVHLSRVLSDLQALVNLLMFATQFRN

>AchiGR5

MSCDALEKSGKKIIKTCYMLHESVGNEHIKEELFLLATYAEQWKPALSAAGFYDLNQTTLSTLFEAIITYLVIIIQFNLALV

>AchiGR6

MSKQEEKLEAKSLFSWLPVPHIRSNALRRTITGLTLIAYFHIMVYYGLPVILLHALAIEIKCHDEIINIAINAKKIPNVLYFRSLNWYFVIIANYFFFGETFAEYLELFIDKYYVVKILFSYHRFFSFCFYFLGMIWFLARLRRKFIRQQFSLLAWTHFLLIIIGFQSYMIVQNIFEGLIWLIIPVCVVILNDVFAYTFGLLLGKTPLISLSPNKTLEGFVFGGISTLILGTFLSFIFCHIPFLICPVRFLETDNGIIMNTNCTPSYLFQPIYYDIGNTGISVKVFPFLLHSLGFSLFASVIAPFGGFCASGFKRAFKVKDFSDLIPGHGGLTDRFDCQFLMATFVNVYIWTFIRIPQVEKIYQKVFQLSEENQLKFYLLLNESLHTRGLMKD

>AchiGR7

MMTNITIGIYGFTSEVLDHGITFSFKEMGLLVDSIYCMVLLYVFCDCSHQTSANIAEGVQLSLMNIKLNSVDVATTREVELFLKAIHLNPPKVSLQGYSVVNRELISSSVGTIAIYLIVLLQFKISLVNLRG

>AchiGR8

MSCDGVEQSGKQIGKICFLHQEPLDKSSLKEELVLFTKITKELAPTFSAAGFFTINQSVLPTLFSTVSTYLIIIIQFNMTL

>AchiGR9

MDLQDINISQENFEILQPIILVARFLGILPVRYDKLGDHFKLKRSLVYSIYSYVLTIFLTVATVLGIVNDLEKDTNHSVRMVDQKARYVTSCDISIVIIIVFFSAVTIPQKMRKLWKLLHYLNQTDSIIPLTRQDQFRQSSLFFMATTFVVAVLLFTFDIVVWTNSTTKRMKDATSFFRNYTTFYVLYLIVMIHEIFYWHLVLFIKIRIAALNRYLHSIGKEDRCKKIPVQAGGRQVVVGGALENIEVCKEGYKKKNFENMSLVERITVLATFQERISNAVQVLNNDGAFGIHLITLSCLLHLIVTPYFLLVEIIKPDGNGMFTYLQAAWLLAHIGRLLIIVEPCQLCLDEHRRTSMLLCELLTKDFDENVRNSLIIFSMQLNYCKIKFSPCGFFKIDRSLITSVTAAVTTYLVILFQFNTN

>AchiGR10

MYLEVDDFIDALNNLCGWPVMLLSFNLVVDILTCLIEAVTWQGQDDVLSSDIALSIWFLVQSVIWLTAIIFCCDSVVRETDELLTNCYRLEQTLPLLSKELEELDSLKKLIKNKRPKLTAAGFFEIRRSTLLSLLSTTTTYFIVALQFNSL

>AchiGR11

MCPHTLDVVSCLYPAYIYSLLMGFAPFKIERKGPFVEIKHCKIYHVLSTLFYTLSICFLVWMGYTKMGYYHLQSPMQRFINFLYLLLVAGLMSVNVLFNKLHTTSLVTLWRKMCHIDTLIRNRGIKLNYRRLMYSTSTFCIVAATLHTCYIIFLVSIQNYKDPLFSGSVYFALNFAVTGFACVSCSFLAFFLLIGHMFEKILEYMKHRVLRSFLLDSDSKSKSIIDTADLHQELCELVRHANDVMSVQMLASFAVTFSVVTLQIYSMVNAFNIKKVDYSFVLSSLCCVIILLQEKVILSLVSRRCMGMIPKTKELLTELLNVHIDDKRICREVNAFNLQILHNQFDITAGNFFVIDPPIFLTAGSAITTYILLFIQADPFVDLYQSMKDSVGAI

>AchiGR12

MQGKAAGGKFSKTKILLVVLAAAGDYVSSEANKTSAICYKVINKIPISSKYDVIKEELKLIAQQTLCRNPRLSAFGFFTVNFTMMGFIVTSITSYIIISIQFLI

>AchiGR13

MFSCDWAIKEAGKLASTCYNYQAYFPTFSVEKQELLNLANQIVNNKPAFTAAGFFEVNCKTLFALFGTTTTYFIVILQFDQM

>AchiGR14

MLNKIKVLVRVYDMLLQASNMTNRNFDIQLVPSLCNQLFLAAYILFYIYWMISEKPYSALCWAIWGILKILEVINFSAACHFASRELHVVGNSIHKVLIHNNNLAIREEVREFFYFIKSNAINLNFSYKSTTSLAYVLFDFS

>AchiGR15

MKLSDVDIILQQYKIATYFGFPLISEVTAPKLKRVCSKWYPFVIFFTYFVFTLNCLTKRISYYIYLFPPMQILNDVLEILSEILFLSVCLLWPTKRFQQWKTFWNNLKTLDKNLKKLNFEEKRNPFIDGTITSVCFVVIFCGHIWEILDWYKIGKIGIIYYYMNFRIVMYYHLFATILICQITQMLKVRFDFLNQYLYKVGKKKHILWDVSQTEAYFTIRQLQEISKLYAMIHGLIKIFNNILGWHICFIIKSTILVILSSITFMVAILSQDIFKRGQISTGLIIINVGYSSIYLVSAY

>AchiGR16

MGKKILVRDVKLIKEVTDRLYHLCRMHYKLCHLMRELNKTFAFQLLCSLAVSMGDVLFQSYYLYHLLFNNVPNVTTTMILCPIAWLVDEMVEIFLLVRSCASSCESANTTPILLHEFRNELDNIEIESHIQMYSLQLLHQKIRVSALGFFDIDYSLIYSIVGAVTTYLVIFIQFDQQTSDIKLYPVGNCSGP

>AchiGR17

MRKNPLVYFIGGCLLYFVSIGCQLFHIGSTVLIYYITPILLHYAKYVLIDIIYNITLSIEIKYKDLNNMLLEINSVTTVTSKSATKLIKKVRILYLKINSVIDTFNSFFGWPLLLLVAHSFAQILTCLSLLATNSYPDVLSARIVISYYLIMTMGFLLRVISGCDNVLKASHNFVQNCFELRESFPLTSKPAEEIEKLQATFKVQSNISAAGFFEINRSLLLSIFSTATTYLIVVIQFRSVYSVQ

>AchiIR1

MLNGYQSKILQINSQNIEYPIPPNRQVFVLNLHCENSSRLLQRVDKLNFFRAPFRWIIYHSYDIETAALAETFFSEINILVDSDVTLCELKTNDTVRIKKLYKNRSNYSINIEEMGYWTKNEGFSDNGFEKNTARRRQDLKGTKLNTCLVITQKNSLYHLTDKRDKHIDSISKVNYVLVEHLKDIINATFNYSVQQTWGYKNKKSEWSGMIGELTRNEADIGGTALFFTIDRINIIDYIAMATPTRSKFVFRQPKLSYVSNVYTLPFDYHVWLCTIFLVIIVGIFLYITVKWEWQKSKHDKMEDTSYPELRDSISDVAVLSFGALC

>AchiIR2

MGLIELVLAGLCLNATCPDESTIESLIISPRQRELTMLAEELKMETLRITTFNNGELSGYRKKGNELIGTGVAFDIIHILQDKYNFNYTIVAPEDNVFLSPTKKLGAKDLLINSRADLIAAFLPIINSFHEEISYSRRFDTAEWVVLMKRPSESATGSGLLAPFTTPVWILIILSLLVVGPIIYLLILIQARLTRDDNNRVYPLPACVWFVYGALLKQGTTINPTTDSSRLLFSTWWIFITILTAFYTANLTAFLTLSKFTLPINEPSDIKQRRYQWVSNRANGIKDYIKEENKEMLPRGAKLADIIGNGTTFPETDDMSILTHYVTDRDMMFIREKSVLDNIMYKDYKEKTKKGIDEVKRCTYVVAKFPITKFPRAFAFRHNFKYTKLFDSAIQHLVESGIIEHKFVENLPDTEICPLNLGNTERQLRNADLLLTYLLVAGGLTVAIVVFILELLIRRYWKKPRRSLRGLLGNNKHVPTPPFENNNNYDNFDYIKKITPPPPSYNSLFQPPFNYRNCQKKNINGRDYWIVKDGRGGRELIPHRTPSALLFQWSR

>AchiIR3

MIFLVSCYLLAMLHLSHEQTTQNLNVLYVNEEGNEVADRAIEVAMTYIKKNNKLGISVDLKRVVGNRTDSNTLLESLCSTYQMMLETQAFPHLVLDTTMTGIGSETVKSFTSALAIPTVSASFGQDGDLRQWRNIDENEKEYLIQICPPADIIPEIVRAIVLNQNITNAAILFDNSFVMDHKYKSLLQNVATRHVITPIRGNGEQKVIEDQLLQLRKLDIVNFFILGSVINIGTVLNAANEISYFNRKFAWHAITQDDGEFKCNCKNASIMFVKPSPNTAFQDRLGTMQRTYQLNVEPIITAAFYLDLALRSFLSIKEMISDGAWKKNNVTNYISCDDYDGKNSPKRMNLNLRKYLSKDSTETPTYGPISVSSNGQSFMEFQMQISAVGVREGASDKSTTLGSWTAGFDNNLTLVEPQVMANMTADVVYRVVTVVQKPFIFKDETAPKKYNGYCIDLIEKIADILKFDYEIVAVDHFGTMDESGKWDGLVKELMEKKADIGLGSMSVMAERENVIDFTVPYYDLVGITILMKLPETPTSLFKFLTVLENEVWLCILAAYFFTSFLMWVFDRWSPYSYQNNREKYKDDEEKREFNLKECLWFCMTSLTPQGGGEAPKNLSGRLVAATWWLFGFIIIASYTANLAAFLTVSRLDTPIESLDDLSKQYKIQYAPLNGSSTQVYFERMANIEARFYEIWKDMSLNDSLSEVERAKLAVWDYPVSDKYTKMWQAMKEAGLPNTLDEAVKKVRDSKSSSEGFAFLGDATDIRYLEATNCDLTVVGEEFSRKPYAIAVQQGSPLKDQFNTAILQLLNRRELERLKEKWWNKNPEKKDCQKADDQSDGISIQNIGGVFIVIFVGIGLACITLAFEYWWYKYRKGSKIIDVQEASHHHHQSKTTFPKDVGFPKGKEGAGNKTVASLYPRPRF

>AchiIR4

MRDTSVEFLKSKYLSSNNRAGLIETSTSTYGIPTSDAKFLATSFEDHVMSKISVTPVKIALQMLSTSAFSSIISMKVVSNLRLVFNVLIPLLYHSTILSCPSSFNRTILTVF
